# Pan-Cancer Analysis of PDIA3: Identifying It as a Potential Biomarker for Tumor Prognosis and Immunotherapy

**DOI:** 10.1155/2022/9614819

**Published:** 2022-08-22

**Authors:** Jing Zhang, Kai Wang, Tuersun Hainisayimu, Hui Li

**Affiliations:** ^1^School of Public Health, Xinjiang Medical University, Urumqi 830017, China; ^2^Department of Medical Engineering and Technology, Xinjiang Medical University, Urumqi 830017, China; ^3^Department of Biochemistry and Molecular Biology, Basic Medicine School, Xinjiang Medical University, Urumqi 830017, China; ^4^Central Laboratory of Xinjiang Medical University, Urumqi 830011, China

## Abstract

Protein disulfide isomerase A3 (PDIA3) is a kind of thiol oxidoreductase with a wide range of functions, and its expression is elevated in a variety of tumors, which is closely related to the invasion and metastasis of tumor cells, and has a significant impact on the immunogenicity of tumor cells. Although more and more studies have shown that PDIA3 plays an important role in the occurrence and development of many tumors, there is no systematic pan-cancer study on PDIA3. Therefore, in this study, the differential expression of PDIA3 in 33 kinds of tumors was analyzed to explore its ability to regulate tumor immunity as a biomarker and evaluate its role in different cancer onset stages or clinical prognosis. In this paper, by analyzing the multilevel data including 33 kinds of cancers in the databases of Cancer Genome Atlas (TCGA), UCSC Xena, Cancer Cell Encyclopedia (CCLE), Genotypic Tissue Expression (GTEx), Human Protein Atlas (HPA), cBioPortal, and GDC; the differential expression level of PDIA3 in different types of malignant tumors and its relationship with prognosis and the potential correlation between PDIA3 expression and microsatellite instability (MSI), tumor mutation load (TMB), mismatch repair gene (MMR), DNA methylation level, and immune infiltration level were analyzed with bioinformatics. The results showed that PDIA3 was highly expressed in 19 types of cancers, but downregulated only in THCA. Next, PDIA3 in different tumors was positively or negatively correlated with patient outcome, Kaplan-Meier survival analysis showed that PDIA3 plays an important role in the prognosis of patients with KIRP, KICH, and CESC and may be used as a prognostic biomarker, and the methylation level of PDIA3 promoter region was closely related to patient outcome in eight tumors. The expression level of PDIA3 was correlated with TMB in 13 tumors and MSI in 9 tumors. Among them, the expression level of PDIA3 in THYM has the strongest correlation with TMB, and the expression level of PDIA3 in READ has the strongest correlation with MSI. In addition, the expression of PDIA3 in eight kinds of tumors, including BRCA, HNSC, THYM, LGG, LUAD, LUSC, PRAD, and THCA, had the highest correlation with the infiltration degree of immune cells, and the expression of PDIA3 had the highest correlation with the infiltration degree of 11 kinds of immune cells, including regulatory T cell and macrophages. And LGG is the tumor most likely to be affected by the tumor microenvironment to affect its development and prognosis. To sum up, this study suggests that PDIA3 plays an important role in the occurrence and development of KIRP, KICH, and CESC and in the immunotherapeutic response of THYM, READ, and LGG and can be used as a prognostic biomarker for these tumors.

## 1. Introduction

Cancer is the main cause of death and is also an important obstacle to prolong life expectancy in all countries of the world. According to GLOBOCAN's global cancer statistics in 2020, there are an estimated 19.3 million new cancer cases and nearly 10 million cancer deaths worldwide. Female breast cancer has surpassed lung cancer as the most common cancer, followed by lung cancer, rectal cancer, prostate cancer, and stomach cancer. Lung cancer remains the main cause of cancer death, followed by rectal cancer, liver cancer, gastric cancer, and female breast cancer [1].

With the establishment of large-scale biological database, the development of high-throughput omics (such as genomics, protein omics, and metabonomics), and the rise of various new detection methods, the ability to trace the origin of tumor has been gradually acquired, which can warn the occurrence of tumor at the early stage of tumor development and meanwhile possibly judge its clinical prognosis [2]. Since tumor biomarkers play an important role in early detection, accurate diagnosis, accurate classification, accurate blocking, and accurate treatment of tumors, and now they have become one of the hot spots in oncology research [3]. In view of the complexity of tumorigenesis, it is crucial to analyze PDIA3 gene and evaluate its correlation with clinical prognosis and potential molecular mechanism.

Protein disulfide isomerase A3 (PDIA3) is a thiol oxidoreductase with a wide range of functions, also known as ERp57, ER60, and GRp58, which is a member of PDI protein family, acting as a chaperone. PDIA3 is highly expressed in cell stress response, blocks apoptotic cell death and protein misfolding related to endoplasmic reticulum stress, and interacts with two lectin-binding chaperonins in endoplasmic reticulum [4]. The expression of PDIA3 is increased in nearly 70% of cancers, with the highest expression in liver, lung, placenta, pancreas, and kidney and the lowest expression in heart, skeletal muscle, and brain [5]. Its expression has low correlation with the invasiveness, survival, and metastasis of the whole cell and has significant influence on the immunogenicity and invasiveness of cancer cells. PDIA3 expression has been proved to be a prognostic biomarker of many cancers, and it is considered as a possible new pharmacological target, which plays a vital role in the occurrence and development of many cancers [6, 7].

Celli and Jaiswal [8] confirmed that the content of transcripts of encoding PDIA3/ERp57 in breast, uterus, lung, and stomach tumors was higher than that in the corresponding normal tissues. Shishkin et al. [9] pointed out that PDIA3 can be used for tumor diagnosis, and it may also lead to the development of chemotherapy. The study by Liu et al. [10] showed that PDIA3 was expressed in many cancers, whose upregulation or downregulation was associated with poor prognosis. The study by Teramoto et al. and Chay et al. [11, 12] have shown that the expression level of PDIA3 in cancer cells is related to the progress and prognosis of some human tumors. The PDIA3 expression is increased in ovarian cancer cells, which is considered as a potential biomarker for the prognosis of ovarian cancer. The expression of PDIA3 in hepatocellular carcinoma is positively correlated with tumor grade and AFP level. The high expression level of PDIA3 is an important potential biomarker for rapid tumor progression and poor prognosis.

However, most studies are limited to the role of PDIA3 in specific tumors, and there is no pan-cancer study of PDIA3 in various tumors. Therefore, by analyzing the multilevel data including 33 cancers in TCGA, UCSC Xena, CCLE, GTEx, HPA, cBioPortal, and other databases, this paper revealed the differential expression of PDIA3 gene in different cancers and explored the important role of PDIA3 gene in the occurrence and development of cancer. At the same time, this study comprehensively analyzed the expression level of PDIA3, its relationship with prognosis, and its potential relationship with tumor immune microenvironment (TME), microsatellite instability (MSI), tumor mutation burden (TMB), DNA methylation level, and immune infiltration level. The paper further conducted coexpression analysis of immune-related genes and mismatch repair genes (MMR) and PDIA3, enrichment analysis of GSEA gene set and variation analysis of GSVA gene set, and in-depth discussion of the role of PDIA3 gene in the pathogenesis of different cancers, which facilitates to understand the role of PDIA3 gene in the occurrence and development of various tumors from a clinical point of view, providing help for clinical diagnosis and treatment and prognosis evaluation, new ideas for the role of PDIA3 in tumor immunotherapy, as well as clues for finding broad-spectrum targets of tumor therapy.

## 2. Methods

### 2.1. Analysis of Differential Expression of PDIA3 in Pan-Cancer

Based on the public database of the cancer genome atlas (TCGA, https://cancergenome.nih.gov/), the gene expression data, somatic mutation data, related clinical data, and phenotypic data of 11,093 samples of 33 cancers are downloaded, and the gene expression data are standardized by log2(TPM + 0.001). Based on the genome annotation information database of GENCODE (https://www.gencodegenes.org/), the annotation files of human genes were downloaded, to lay the foundation for the next gene ID transformation, and the gene expression data of PDIA3 in 33 cancers were extracted by R software. Based on UCSC Xena (https://xena.ucsc.edu/) cancer genomics database, RNAseq data of 11,057 samples of 33 cancers were downloaded, and the data were standardized as log2(FPKM + 1) type. The data of 24 tumor cell lines were downloaded from CCLE database (https://portals.broadinstitute.org/ccle/), and the gene expression data of 31 different normal tissues were downloaded from GTEx database (https://commonfund.nih.gov/GTEx). The differential expression of PDIA3 in 33 cancers in TCGA database was analyzed, and these tumor types underwent Wilcoxon rank sum test. The differential expression level between tumor tissue samples and normal tissue samples in 33 cancers was compared. *P* < 0.05 was considered to be different in tumor tissue and normal tissue, and the distribution of gene expression level was shown by box diagram. The expression of PDIA3 in 24 tumor cell lines, 31 normal tissues, and 33 tumor tissues was further analyzed. The R software (version 4.0.5; https://www.R-project.org) was used for data processing and analysis, and R software package “ggpubr” was used to plot box diagram. This paper follows the research method of Yao et al. 2022 [13].

### 2.2. Immunohistochemical Staining Analysis

The immunohistochemical images of PDIA3 protein expression in normal tissues and eleven tumor tissues were downloaded based on the database of Human Protein Atlas (HPA) (https://www.proteinatlas.org/), including breast invasive carcinoma (BRCA), colon adenocarcinoma (COAD), esophageal carcinoma (ESCA), kidney renal clear cell carcinoma (KIRC), liver hepatocellular carcinoma (LIHC), lung adenocarcinoma (LUAD), lung squamous cell carcinoma (LUSC), prostate adenocarcinoma (PRAD), stomach adenocarcinoma (STAD), thyroid carcinoma (THCA), and uterine corpus endometrial carcinoma (UCEC). The protein expression of PDIA3 in these eleven tumors was analyzed, and the differential expression of PDIA3 in normal tissue samples and tumor tissue samples was also analyzed. The R software package “ggpubr” and “ggplot2” were used to plot a box diagram [13].

### 2.3. Analysis of the Relationship between PDIA3 Expression and Prognosis and Clinical Phenotype

After the samples with incomplete survival information and survival time less than 30 days were excluded, the survival data and clinical phenotype data of each sample of 33 kinds of cancer patients downloaded from TCGA public database were extracted and matched to finally obtain 9,892 cancer patients for total survival time (OS), 9,457 cancer patients for disease-specific survival time (DSS), 5,206 cancer patients for disease-free interval (DFI), and 9714 cancer patients for the progression-free interval (PFI). Four survival indexes, OS, DSS, DFI, and PFI, were selected to analyze the relationship between the expression of PDIA3 and the prognosis of the patients with 33 cancers. The Kaplan-Meier method and log-rank test were used for survival analysis (*P* < 0.05), and R software packages “survival” and “survminer” were used to plot survival curves. In addition, Cox proportional hazard regression model was analyzed by using R software package “survival” and “forestplot” to further determine the relationship between PDIA3 expression and survival prognosis in pan-cancer. Two clinical phenotypes of 33 cancers, tumor stage and patient age, were selected to explore their relationship with PDIA3 expression. Cancer patients were divided into two groups, with 65 years as the critical value. The R software package “limma” and “ggpubr” were used to analyze the correlation between the two selected clinical phenotypes, and the difference was considered to be significant (*P* < 0.05) [13].

### 2.4. Correlation Analysis of PDIA3 Expression with Tumor Mutation Load, Microsatellite Instability, and Mismatch Repair Genes

In this study, according to somatic mutation data of 33 cancer types downloaded from TCGA (https://tcga.xenahubs.net) database, Perl script was used to calculate the tumor mutational burden (TMB) score, which was corrected by dividing the total length of exons to get the TMB data of 33 cancer types. The MSI data of 33 cancers were downloaded from cBioPortal database, and the relationship between PDIA3 expression and TMB and MSI was analyzed by Spearman rank correlation coefficient. Based on the expression data of mismatch repair genes (MMR) in different cancers in TCGA database, including the expression data of epithelial cell adhesion molecule (EPCAM), postmeiotic segregation increased by 2 (PMS2), MSH6, MSH2, and MLH1, the correlation between MMR gene expression level and PDIA3 expression level was discussed. R software package “fmsb” was used to generate correlation radar map for data visualization, and R software packages “reshape2” and “RColorBrewer” were used to plot heat map.

### 2.5. Relationship between PDIA3 Expression Level and Tumor Immune Microenvironment

In this study, the gene expression data of 33 cancer samples in TCGA public database were used to estimate stromal cells and immune cells in malignant tumor tissues and then to infer the degree of tumor infiltration by stromal cells or immune cells. The ESTIMATE algorithm was used to calculate the immune score and matrix score of 33 cancer samples. The R software package “estimate” and “limma” were used to evaluate the relationship between PDIA3 expression and immune score and matrix score in 33 cancers according to the degree of immune infiltration, and if *P* < 0.05, the difference was considered to be statistically significant. The R software package “ggplot2,” “ggpubr,” and “ggExtra” were used to visualize the correlation analysis results.

### 2.6. Correlation Analysis between PDIA3 Expression Level and Tumor Immune Cell Infiltration Level

The study is based on the relative scores of 26 immune cells in 33 cancers calculated by CIBERSORT, a metagene tool that can predict the phenotype of immune cells, downloaded from the cancer genome data sharing GDC database (https://gdc.cancer.gov/about-data/publications/panimmune) of National Cancer Institute (NCI). The infiltration of nontumor cells was predicted by analyzing the specific gene expression characteristics of immune cells and stromal cells. The R package “ggplot2,” “ggpubr,” and “ggExtra” were used to evaluate the correlation between the expression level of PDIA3 and the infiltration level of immune cells in 33 cancers, and *P* < 0.05 is considered to be significantly different. In addition, immune-related genes were downloaded from IMMPORT (https://www.immport.org/shared/home) immune gene set database, and the R package “limma” was used to conduct coexpression analysis on PDIA3 and immune-related genes (including genes encoding major histocompatibility complex (MHC), chemokines, and chemokine receptor proteins), and the R package “reshape2” and “RColorBrewer” were used to generate coexpression heat map for data visualization [13].

### 2.7. Correlation Analysis between PDIA3 Expression and DNA Methylation

In this study, HM450 methylation data downloaded from UCSC Xena (https://xena.ucsc.edu/) cancer genomics database and annotation file of illuminaMethyl450_hg38_GDC.GDC methylation probe were downloaded, and then, the correlation between PDIA3 expression and gene promoter methylation in 33 cancers was analyzed. Kaplan-Meier survival analysis was used to analyze the correlation between methylation of PDIA3 gene promoter and clinical prognosis (including OS, DSS, DFI, and PFI). *P* < 0.05 was considered to be significantly different [13].

### 2.8. Gene Set Enrichment Analysis and Gene Set Variation Analysis of PDIA3 Expression in Various Tumors

Based on gene set enrichment analysis (GSEA) and gene set variation analysis (GSVA), the biological function and significance of PDIA3 in 33 cancers were studied. Gene ontology (GO) c5.all.v7.4.symbols.gmt gene set and Kyoto Encyclopedia of Genes and Genomes (KEGG) C2.cp.Kegg.v7.4.symbols.gmt gene set were downloaded from GSEA (http://www.gsea-msigdb.org/gsea/index.jsp) database. R software packages “limma,” “org.Hs.eg.db,” “clusterProfiler,” and “enrichplot” were used for GO functional annotation and KEGG pathway analysis of 33 cancers, and R software packages “colorspace,” “stringi,” and “ggplot2” were used to visually display the results of functional enrichment analysis. The file msigdb.v7.4.symbols.gmt of GSVA gene set was downloaded from MSigDB (http://www.gsea-msigdb.org/gsea/downloads.jsp) gene set database, and GSVA analysis of eight cancer types was carried out according to the results of GO and KEGG analysis. R package “GSVA,” “GSEABase,” and “GSVAdata” were used to obtain the GSVA scores of eight cancers, and R package “future.apply” was used for correlation test. The correlation between PDIA3 expression and more than 25,746 pathways in each tumor was analyzed, and R package “ggpubr” was used to visually generate lollipop charts to display the 10 most significant positive and negative correlation pathways.

### 2.9. Statistical Analysis

In this study, all gene expression RNAseq data underwent log2 standardization, and Wilcoxon rank sum test was used to compare the differential expression of PDIA3 between normal tissue samples and tumor tissue samples in 33 cancers, and if *P* < 0.05, the difference was considered to be statistically significant (*P* < 0.05). Kaplan-Meier method, log-rank test, and Cox proportional hazard regression model were used for clinical prognosis analysis. Spearman test or Pearson test was used for correlation analysis between the two variables, *P* < 0.05 was significantly different, and all statistical analysis was made by R software (version 4.0.5; http://www.R-project.org) for data processing.

## 3. Results

### 3.1. Analysis of Differential Expression of PDIA3 in Tumor and Normal Tissue Samples

**Figure 1:**
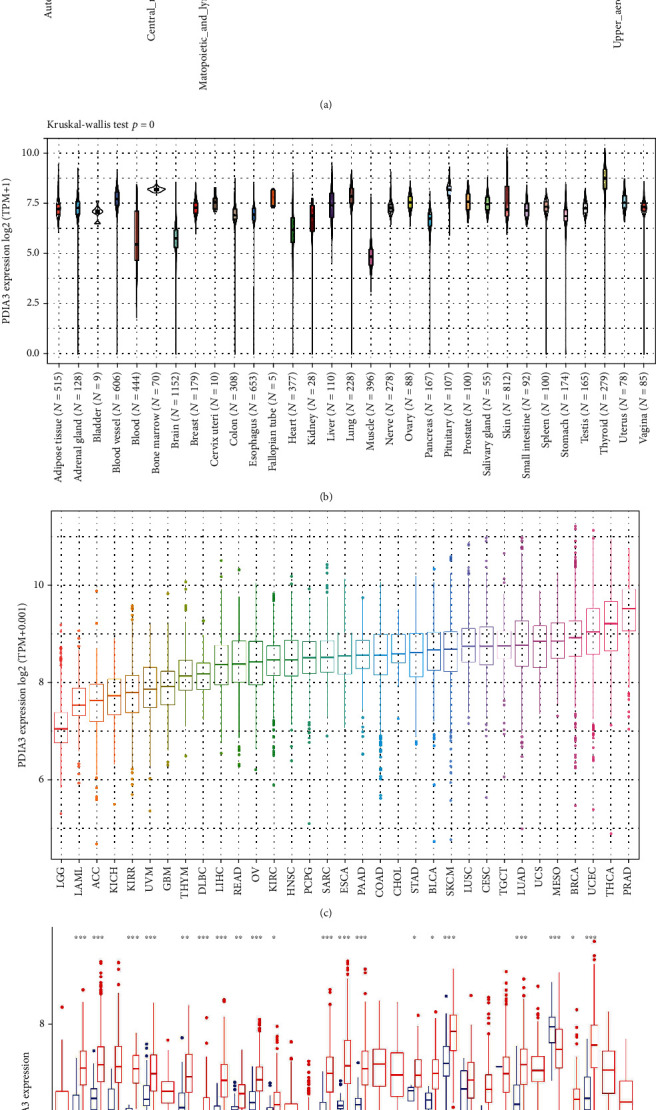
(a) Expression of PDIA3 in 24 tumor cell lines. (b) Expression of PDIA3 in 31 normal tissues. (c) Expression of PDIA3 in 33 cancers. (d) Differential expression of PDIA3 in tumor tissue samples and normal tissue samples of 33 cancers (^∗^*P* < 0.05, ^∗∗^*P* < 0.01, and ^∗∗∗^*P* < 0.001).

The relative expression level of PDIA3 in 24 different tumor cell lines was analyzed based on the data downloaded from the CCLE database of cancer cell encyclopedia, and the expression level of PDIA3 in most normal cell lines was higher (*P* < 0.001) (Figure 1(a)). The expression level of PDIA3 in 31 normal tissues was analyzed based on the data downloaded from the GTEx database, and the expression level of PDIA3 in most normal tissues was higher. Among them, the expression level of PDIA3 was the highest in thyroid tissue (*P* = 0) (Figure 1(b)). It is found in the study that PDIA3 was expressed in all types of tumors, with the lowest expression in brain lower grade glioma (LGG) and the highest expression in prostate adenocarcinoma (PRAD) in order of expression level from lowest to highest (Figure 1(c)).

Based on the data downloaded from the TCGA database, the expression levels of PDIA3 in tumor tissues and matched normal tissues of 33 kinds of cancers were analyzed (Figure 1(d)). It was found that there were significant differences in PDIA3 expression between tumor tissues and normal tissues among 20 types of cancers. Among them, PDIA3 was highly expressed in bladder urothelial carcinoma (BLCA), breast invasive carcinoma (BRCA), cholangiocarcinoma (CHOL), colon adenocarcinoma (COAD), esophageal carcinoma (ESCA), glioblastoma multiforme (GBM), head and neck squamous cell carcinoma (HNSC), kidney chromophobe (KICH), kidney renal clear cell carcinoma (KIRC), kidney renal papillary cell carcinoma (KIRP), liver hepatocellular carcinoma (LIHC), lung adenocarcinoma (LUAD), lung squamous cell carcinoma (LUSC), pancreatic adenocarcinoma (PAAD), pheochromocytoma and paraganglioma (PCPG), prostate adenocarcinoma (PRAD), stomach adenocarcinoma (STAD), thymoma (THYM), and uterine corpus endometrial carcinoma (UCEC). On the contrary, compared with normal tissues, the expression of PDIA3 in thyroid carcinoma (THCA) is downregulated. Among them, the expression of PDIA3 in glioblastoma multiforme (GBM) showed the most significant difference between tumor and normal tissues. There was no significant difference between the expression of PDIA3 in tumor tissue and normal tissue among cervical squamous cell carcinoma and endocervical adenocarcinoma (CESC), rectum adenocarcinoma (READ), sarcoma (SARC), and skin cutaneous melanoma (SKCM). There was no significant difference in the expression level of PDIA3 in the cancers with only normal tissue samples, including adrenocortical carcinoma (ACC), lymphoid neoplasm diffuse large B-cell lymphoma (DLBC), acute myeloid leukemia (LAML), brain lower grade glioma (LGG), mesothelioma (MESO), ovarian serous cystadenocarcinoma (OV), testicular germ cell tumors (TGCT), uterine carcinosarcoma (UCS), and uveal melanoma (UVM).

### 3.2. PDIA3 Differentially Expressed Protein Level in Different Tumors

**Figure 2:**
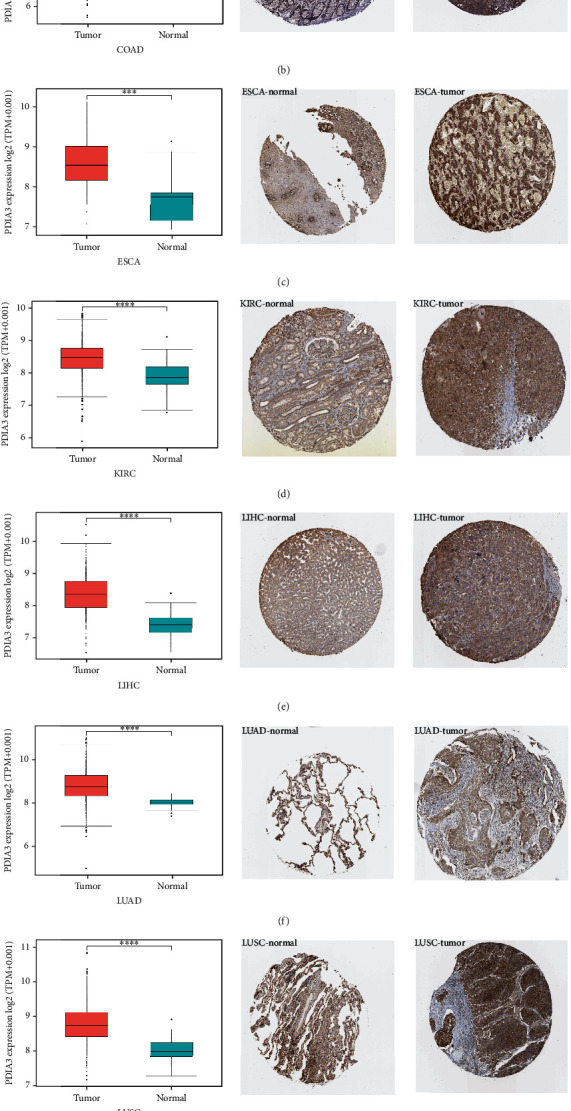
Expression of PDIA3 in tumor tissues and normal tissues of different cancers in TCGA database (left), immunohistochemical image of PDIA3 in normal tissues in HPA database (middle), and immunohistochemical image of PDIA3 in tumor tissues in HPA database (right). (a) Breast. (b) Colon. (c) Esophagus. (d) Kidney. (e) Liver. (f, g) lung. (h) prostate (^∗^*P* < 0.05, ^∗∗^*P* < 0.01, and ^∗∗∗^*P* < 0.001).

In order to further evaluate the expression of PDIA3 protein level, the IHC results in HPA database were analyzed, which was compared with the results of differential expression analysis of PDIA3 between tumor tissues and normal tissues in TCGA database, as shown in (Figures 2(a)–2(h)), the results for other cancers are shown in Supplementary Figures s1. The data analysis results of PDIA3 in these two databases were consistent with each other. PDIA3 showed moderate IHC staining in normal bladder, colon, esophagus, lung, prostate, and stomach tissues, but strong staining in tumor tissues. PDIA3 showed weak staining in normal breast, kidney, liver, and uterus tissues, but moderate staining in tumor tissues. On the contrary, PDIA3 showed moderate staining in normal thyroid tissues, but weak staining in tumor tissues.

### 3.3. Analysis of the Relationship between PDIA3 Expression and Prognosis in Different Tumors

**Figure 3:**
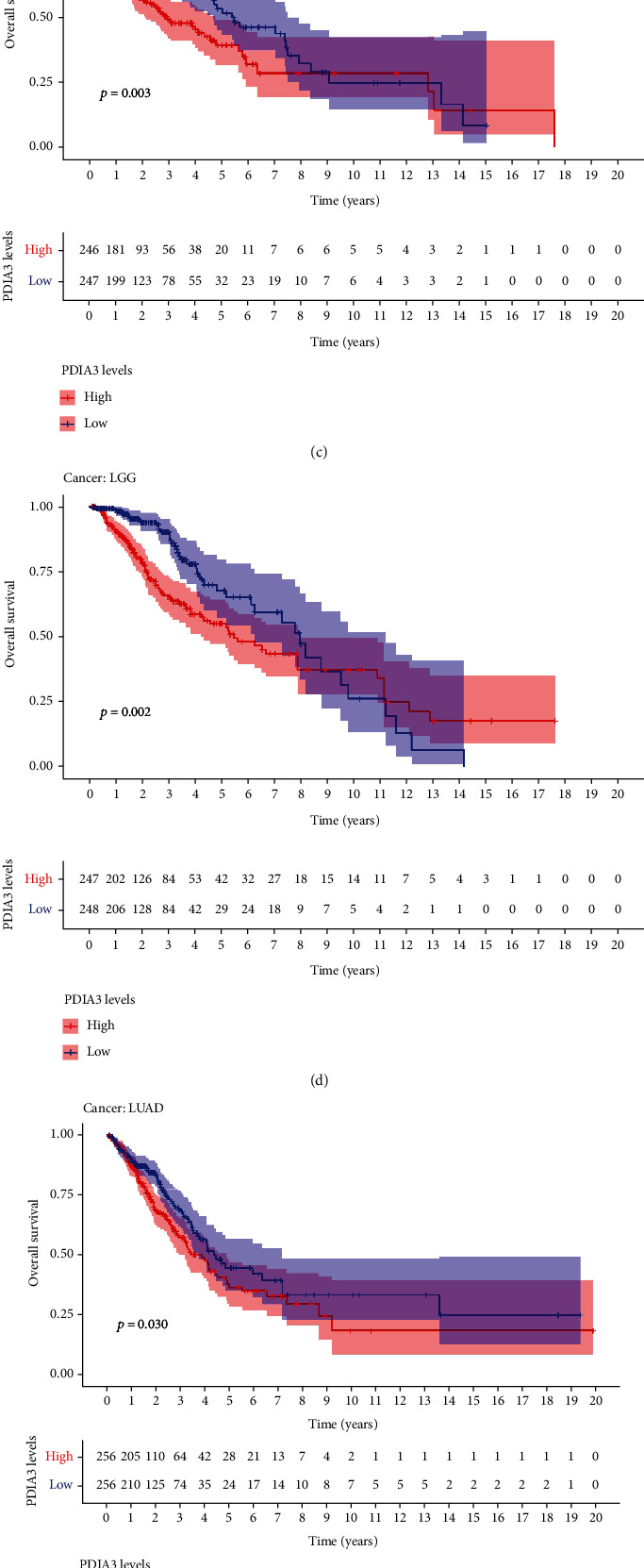
Correlation between PDIA3 expression and total survival time (OS). (a) Forest map of OS correlation in 33 tumors. (b–g) Survival analysis between PDIA3 expression and OS.

In order to explore the relationship between PDIA3 expression level and patient outcomes, the survival of 33 tumors was analyzed in this study, including four outcome indicators OS, DSS, DFI, and PFI. Cox proportional risk regression model showed that PDIA3 expression in ACC (*P* = 0.011), CESC (*P* = 0.006), GBM (*P* = 0.003), HNSC (*P* = 0.004), KICH (*P* = 0.003), KIRP (*P* = 0.001), LAML (*P* = 0.022), LGG (*P* < 0.001), UCEC (*P* = 0.05), and UVM (*P* = 0.002) was associated with OS of tumor patients (Figure 3(a)). The results of OS analysis suggest that PDIA3 is a high-risk gene for ACC, CESC, GBM, HNSC, KICH, KIRP, LGG, and UVM, especially KICH (hazard ratio = 1.009), while it is a low-risk gene for LAML and UCEC. The results of Kaplan-Meier survival analysis showed that among patients with CESC (*P* = 0.014) (Figure 3(b)), HNSC (*P* = 0.003) (Figure 3(c)), LGG (*P* = 0.002) (Figure 3(d)), LUAD (*P* = 0.030) (Figure 3(e)), and UVM (*P* = 0.004) (Figure 3(f)), patients with high expression of PDIA3 had a short overall survival time and poor prognosis, while among patients with UCEC (*P* = 0.002) (Figure 3(g)), patients with high expression of PDIA3 had a long overall survival time and good prognosis.

**Figure 4:**
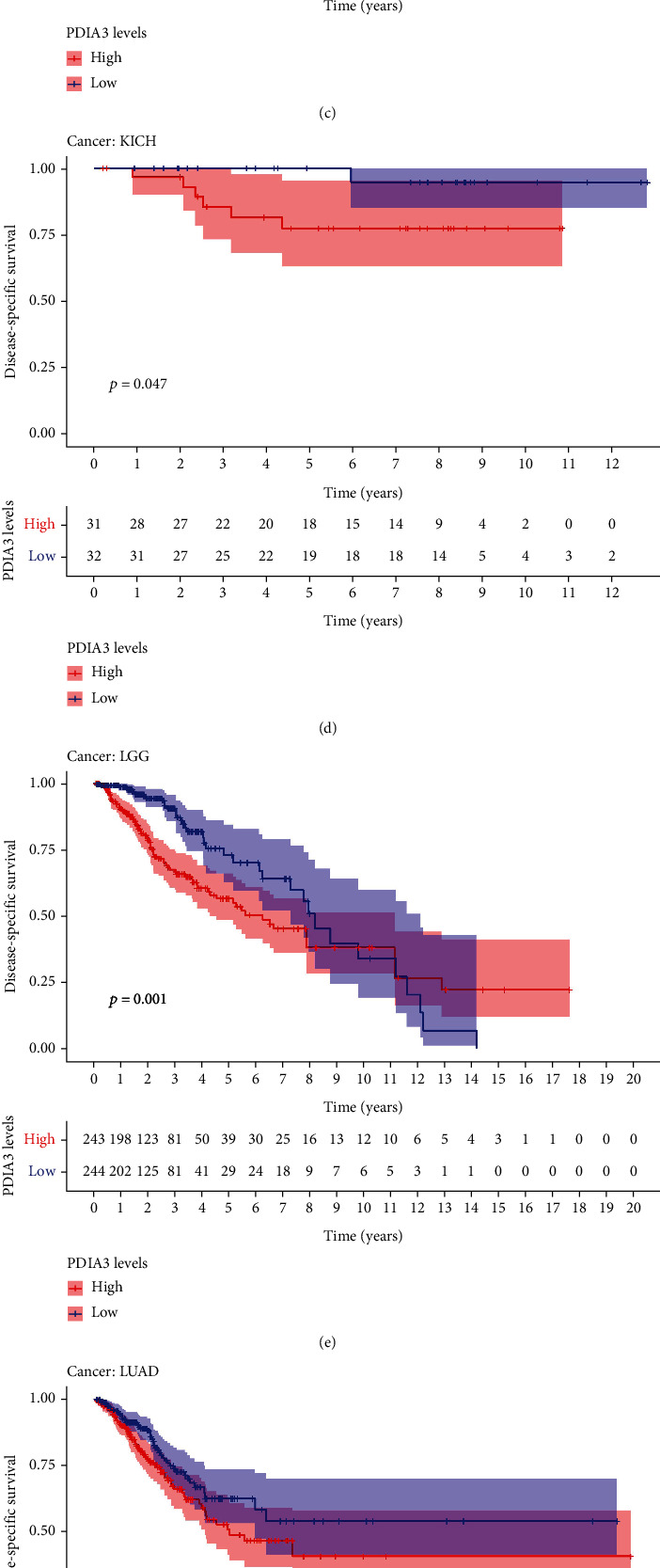
Correlation between PDIA3 expression and disease-specific survival (DSS). (a) Forest map of DSS correlation in 33 tumors. (b–h) Survival analysis between PDIA3 expression and DSS.

The results of DSS data analysis showed that in GBM (*P* = 0.004), HNSC (*P* = 0.007), KICH (*P* = 0.005), KIRC (*P* = 0.002), KIRP (*P* < 0.001), and LGG (*P* < 0.001) patients, the high expression of PDIA3 was correlated with poor outcomes, and UVM (*P* = 0.002) patients (Figure 4(a)), especially KICH (hazard ratio = 1.01). The results of Kaplan Meier survival analysis showed that in CESC (*P* = 0.013) (Figure 4(b)), HNSC (*P* = 0.009) (Figure 4(c)), KICH (*P* = 0.047) (Figure 4(d)), LGG (*P* = 0.001) (Figure 4(e)), LUAD (*P* = 0.046) (Figure 4(f)), and UVM (*P* = 0.003) (Figure 4(g)) patients, the high expression of PDIA3 was correlated with poor outcome, while in UCEC (*p* = 0.012) patients (Figure 4(h)), the high expression of PDIA3 was correlated with good patient outcomes.

**Figure 5:**
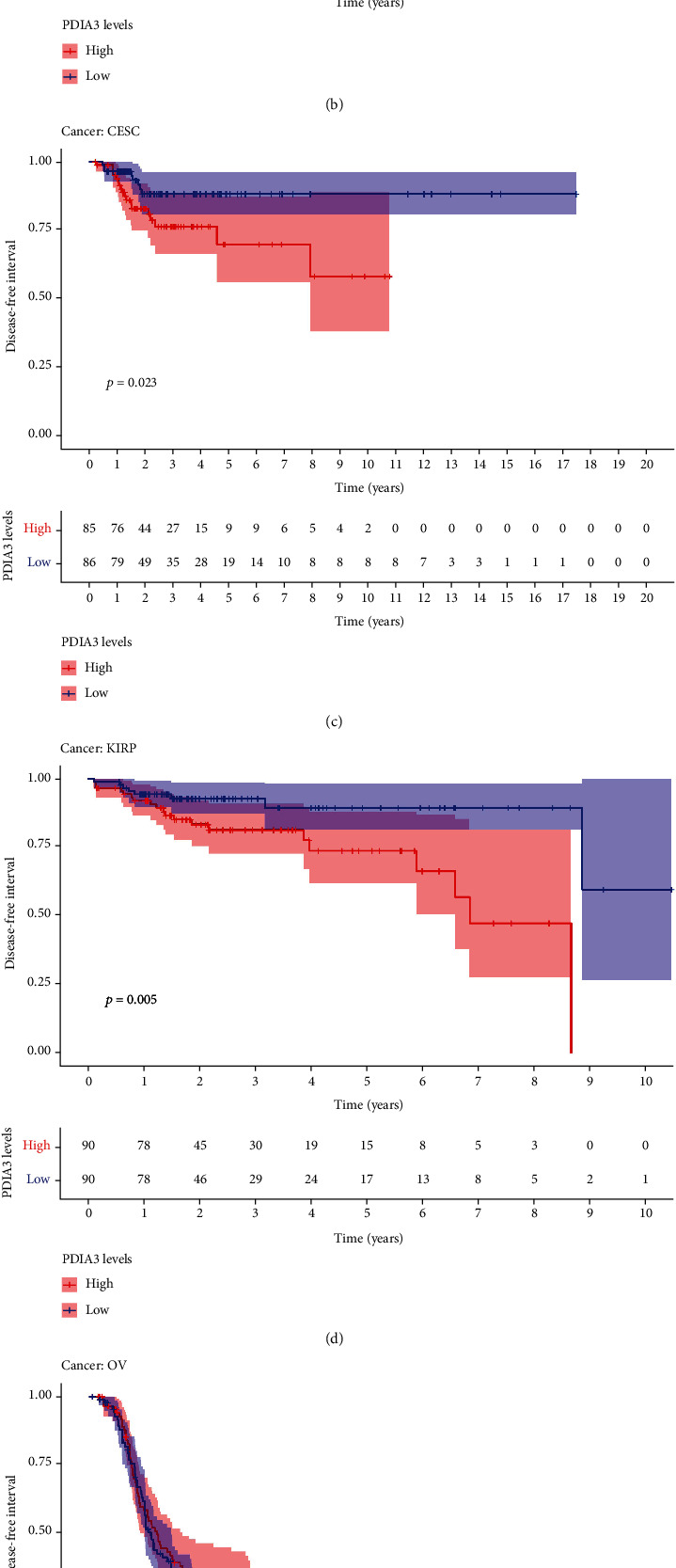
Correlation between PDIA3 expression and disease-free interval (DFI). (a) Forest map of DFI correlation in 33 tumors. (b–f) Survival analysis between PDIA3 expression and DFI.

The results of DFI data analysis showed that in ACC (*P* = 0.004), CESC (*P* = 0.006), COAD (*P* = 0.02), ESCA (*P* = 0.021), KIRP (*P* < 0.001), and LUSC (*P* = 0.008) patients, PDIA3 expression was associated with DFI (Figure 5(a)). DFI analysis showed that PDIA3 was a high-risk gene for ACC, CESC, COAD, KIRP, and LUSC, especially ACC (hazard ratio = 1.01), while PDIA3 was a low-risk gene for ESCA. Kaplan-Meier survival analysis showed that in ACC (*P* < 0.001) (Figure 5(b)), CESC (*P* = 0.023) (Figure 5(c)), and KIRP (*P* = 0.005) (Figure 5(d)), the high expression of PDIA3 was associated with poor patient outcomes, while in OV (*P* = 0.026) (Figure 5(e)) and THCA (*P* = 0.046) (Figure 5(f)), the high expression of PDIA3 was associated with good patient outcomes.

**Figure 6:**
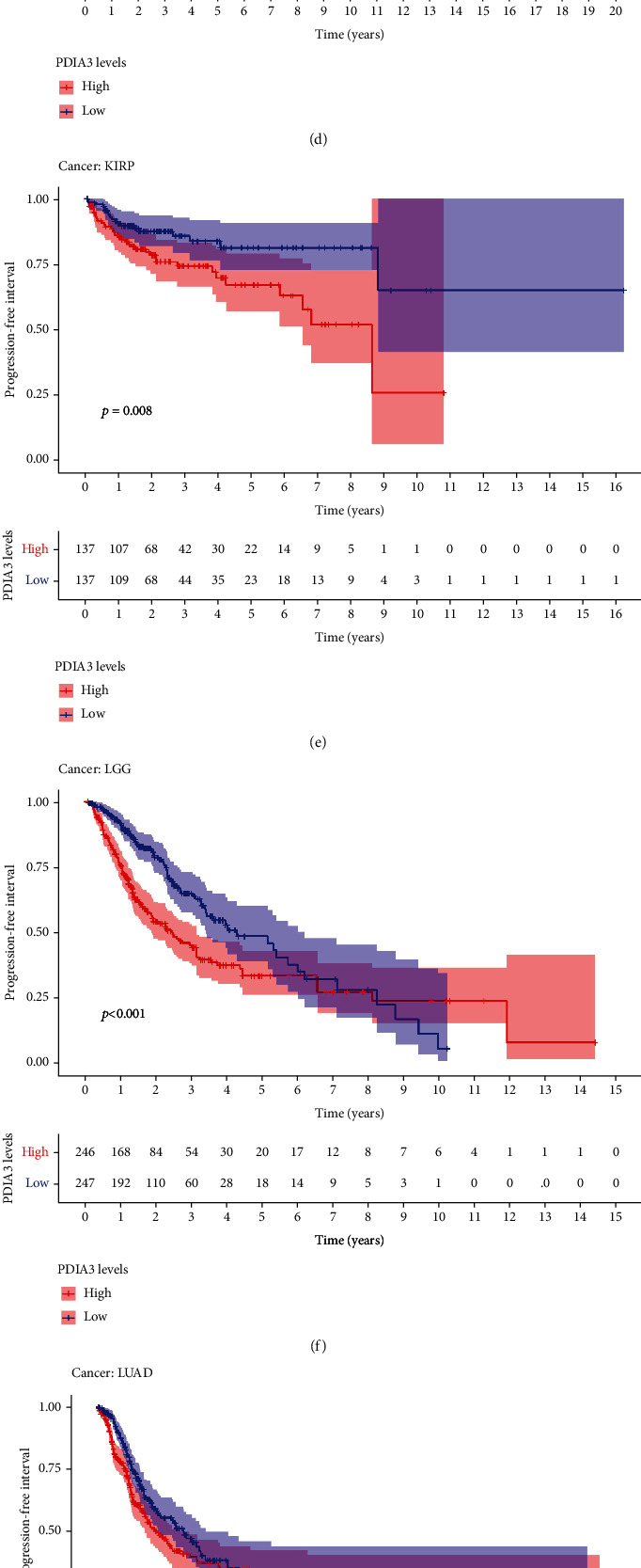
Correlation between PDIA3 expression and progression-free interval (PFI). (a) Forest map of PFI correlation in 33 tumors. (b–j) Survival analysis between PDIA3 expression and PFI.

The relationship between PDIA3 expression and PFI was further analyzed, and the results showed that high expression of PDIA3 in CESC (*P* < 0.001), HNSC (*P* = 0.003), KICH (*P* = 0.039), KIRC (*P* < 0.001), KIRP (*P* < 0.001), LGG (*P* < 0.001), and UVM (*P* < 0.001) was associated with poor PFI. However, in PRAD (*P* = 0.033), the low expression of PDIA3 was associated with poor PFI (Figure 6(a)), especially KICH (hazard ratio = 1.006). Kaplan-Meier survival analysis results showed that in ACC (*P* = 0.015) (Figure 6(b)), CESC (*P* = 0.001) (Figure 6(c)), HNSC (*P* = 0.015) (Figure 6(d)), KIRP (*P* = 0.008) (Figure 6(e)), LGG (*P* < 0.001) (Figure 6(f)), LUAD (*P* = 0.043) (Figure 6(g)), and UVM (*P* = 0.002) (Figure 6(h)), the high-expression PDIA3 was associated with poor PFI, whereas in PRAD (*P* = 0.028) (Figure 6(i)) and THCA (*P* = 0.001) (Figure 6(j)), patients with high expression of PDIA3 had a long survival time.

In conclusion, the results of OS, DSS, DFI, and PFI showed that PDIA3 was a high-risk factor for KIRP, and the results of OS, DSS, and PFI analyses were highly consistent, indicating that PDIA3 was the high-risk gene with the greatest risk of KICH, and the hazard ratio was 1.009, 1.01, and 1.006. Kaplan-Meier curve analysis of all four outcome indicators suggested that higher PDIA3 expression in CESC was associated with poorer survival outcomes, indicating the important role of PDIA3 in the prognosis of KIRP, KICH, and CESC patients and its potential as a prognostic biomarker.

### 3.4. Relationship between PDIA3 and Clinical Features in Different Tumors

**Figure 7:**
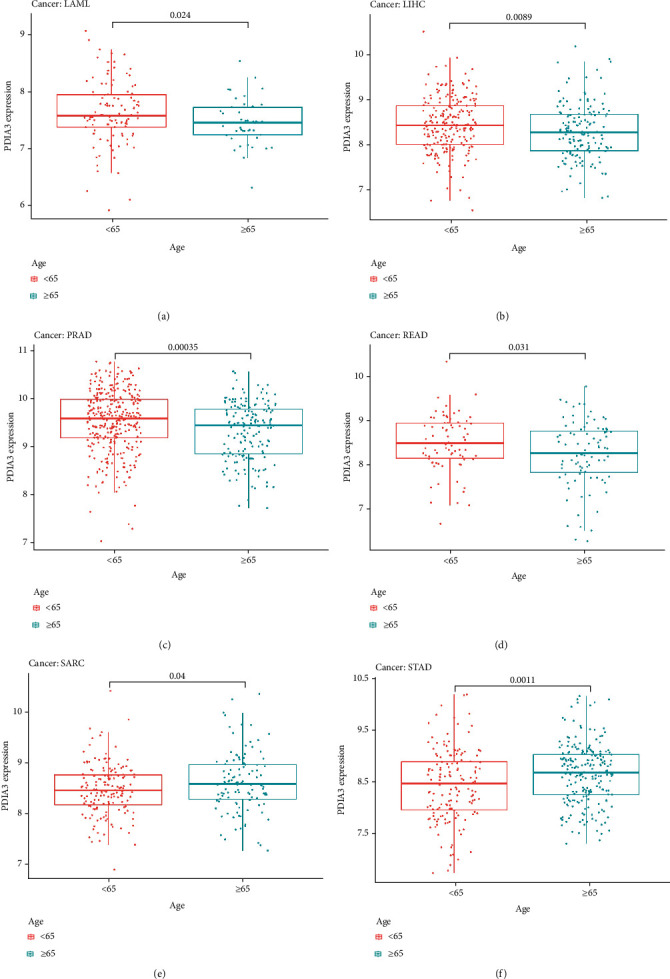
Differential expression of PDIA3 among different age groups in various tumors. (a) LAML. (b) LIHC. (c) PRAD. (d) READ. (e) SARC. (f) STAD.

To explore the relationship between PDIA3 expression and clinicopathological features, the differential expression of PDIA3 among different age groups was analyzed. The results showed the expressions of PDIA3 in LAML (*P* = 0.024) (Figure 7(a)), LIHC (*P* = 0.0089) (Figure 7(b)), PRAD (*P* < 0.001) (Figure 7(c)), and READ (*P* = 0.031) (Figure 7(d)); the expression level of PDIA3 was high in patients aged < 65, while in SARC (*P* = 0.04) (Figure 7(e)) and STAD (*P* = 0.001) (Figure 7(f)), the expression level of PDIA3 was high in patients aged ≥ 65 years.

**Figure 8:**
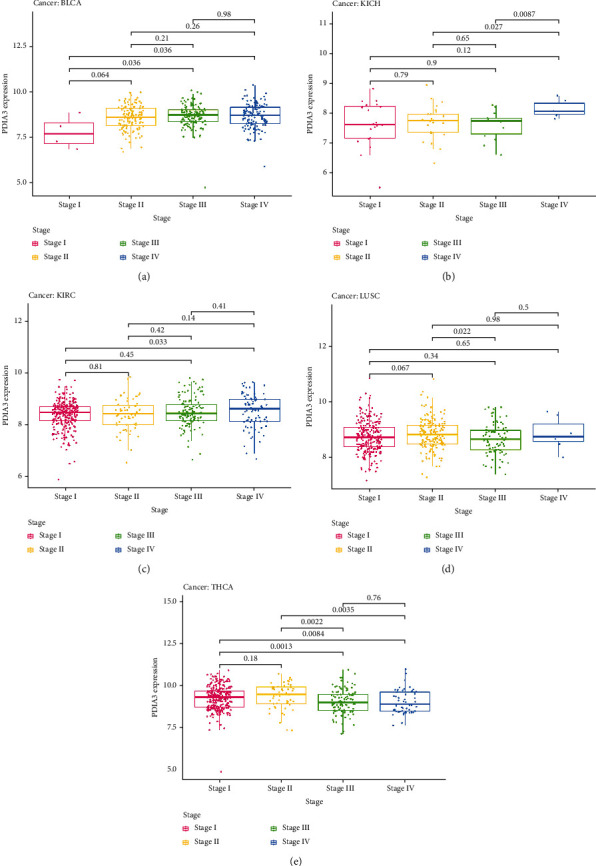
Differential expression of PDIA3 in different clinical stages of various tumors. (a) BLCA. (b) KICH. (c) KIRC. (d) LUSC. (e) THCA.

At the same time, this study analyzed the expression of PDIA3 in different clinical stages (I, II, III, and IV). The results showed that the expression of PDIA3 in different clinical stages of five kinds of tumors was statistically significant, including BLCA, KICH, KIRC, LUSC, and THCA. In BLCA, the expression of PDIA3 increased in stage I and III (*P* = 0.036) and stage I and IV (*P* = 0.036) tumors (Figure 8(a)). In KICH, the expression of PDIA3 increased in stage II and IV (*P* = 0.027) and stage III and IV (*P* = 0.0087) tumors (Figure 8(b)). In KIRC, the expression of PDIA3 increased in stage I and IV tumors (*P* = 0.033) (Figure 8(c)). In LUSC, the expression of PDIA3 increased in stage II and III tumors (*P* = 0.022) (Figure 8(d)). In THCA, the expression of PDIA3 decreased in stage I and III (*P* = 0.0013), stages I and IV (*P* = 0.0084), stages II and III (*P* = 0.0022), stages II and IV (*P* = 0.0035) tumors (Figure 8(e)).

### 3.5. Correlation Analysis between PDIA3 Expression Level and TMB, MSI, and MMR

**Figure 9:**
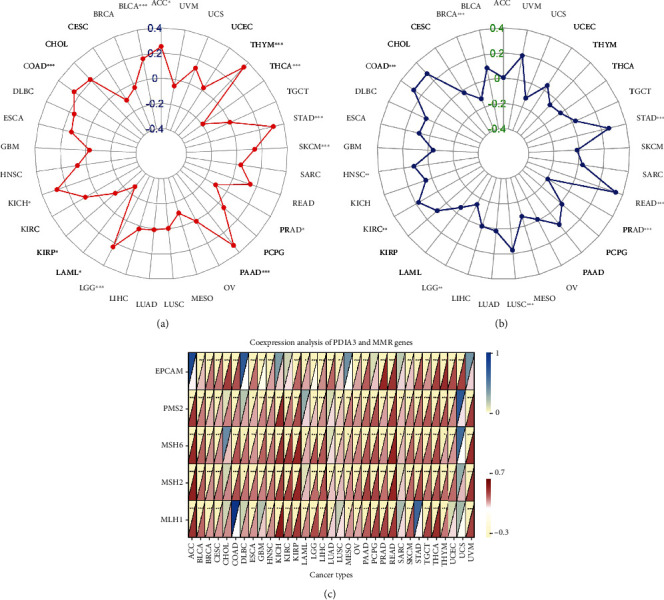
(a) Correlation between PDIA3 expression and tumor mutation load (TMB). (b) Correlation between PDIA3 expression and microsatellite instability (MSI). (c) Correlation between PDIA3 expression and mismatch repair gene (MMR) (^∗^*P* < 0.05, ^∗∗^*P* < 0.01, and ^∗∗∗^*P* < 0.001).

In this study, the correlation between PDIA3 expression level and TMB, MSI, and MMR was further discussed. TMB and MSI were closely related to the sensitivity of tumor immune checkpoint inhibitors. The results showed that the expression of PDIA3 in 13 tumors including ACC, BLCA, COAD, KICH, KIRP, LAML, LGG, PAAD, PRAD, SKCM, STAD, THCA, and THYM was related to TMB (Figure 9(a)). There is a significant positive correlation between PDIA3 gene expression and tumor mutation load in 9 cancers, including ACC, BLCA, COAD, KICH, LGG, PAAD, SKCM, STAD, and THYM, and the correlation coefficient with THYM is the highest (cor = 0.35). There is a significant negative correlation between PDIA3 gene expression and tumor mutation load in KIRP, LAML, PRAD, and THCA in the other 4 cancers. The expression of PDIA3 was correlated with MSI in 9 tumors including BRAC, COAD, HNSC, KIRC, LGG, LUSC, PRAD, READ, and STAD (Figure 9(b)). There is a significant positive correlation between PDIA3 gene expression and microsatellite instability in 6 cancers, including COAD, HNSC, KIRC, LUSC, READ, and STAD, and the correlation coefficient with READ is the highest (cor = 0.342). There is a significant negative correlation between PDIA3 gene expression and microsatellite instability in the other three cancers BRCA, LGG, and PRAD. The analysis results of the correlation between the expression level of PDIA3 and MMR mismatch repair genes EPCAM, PMS2, MSH6, MSH2, and MLH1 showed that the expression level of PDIA3 was positively correlated with the expression level of MMR gene in most tumors, but it was not correlated with EPCAM gene in ACC, DLBC, and MESO, and not correlated with MLH1 in COAD and STAD, and was only strongly correlated with EPCAM gene in UCS (Figure 9(c)).

### 3.6. Correlation Analysis between PDIA3 Expression and Tumor Immune Microenvironment

**Figure 10:**
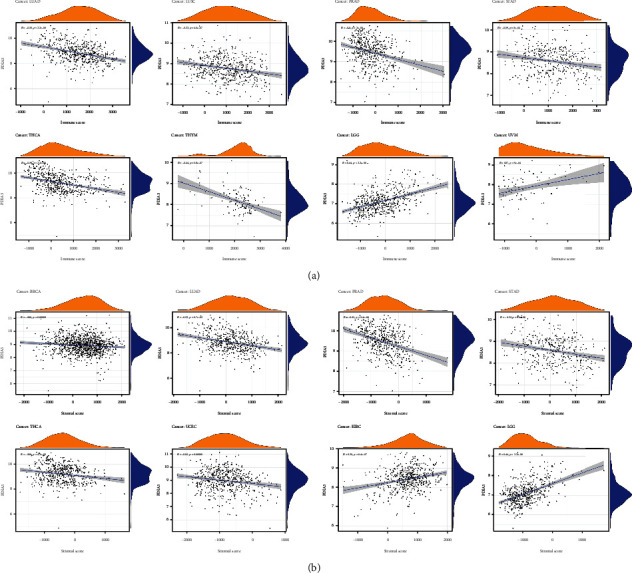
(a) Correlation between PDIA3 expression and immune score in each tumor. (b) Correlation between PDIA3 expression and matrix score in each tumor.

The immune microenvironment of tumor is closely related to its occurrence and development. Therefore, the relationship between PDIA3 expression and TME was further explored in the study. The ESTIMATE algorithm was used to calculate the immune score and matrix score of 33 different tumors, and the relationship between PDIA3 expression and these two scores was analyzed. The results showed that PDIA3 was negatively correlated with immune score in LUAD, LUSC, PRAD, STAD, THCA, and THYM, but positively correlated with immune score in LGG and UVM (Figure 10(a)) and that PDIA3 was negatively correlated with matrix score in BRCA, LUAD, PRAD, STAD, THCA, and UCEC and positively correlated with matrix score in KIRC and LGG (Figure 10(b)). Among them, in LGG, the correlation coefficient between PDIA3 gene expression and immune score and matrix score is the highest and has a significant positive correlation.

### 3.7. Relationship between the Expression of PDIA3 and the Infiltration Level of Tumor Immune Cells

The relationship is between the expression level of PDIA3 and the infiltration level of 26 kinds of immune cells. The results showed that the expression of PDIA3 was related to the infiltration level of immune cells in most types of tumors. There were 8 tumors, including BRCA (*n* = 16), HNSC (*n* = 15), THYM (*n* = 15), LGG (*n* = 14), LUAD (*n* = 15), LUSC (*n* = 13), PRAD (*n* = 13), and THCA (*n* = 13), in which the expression of PDIA3 had the highest correlation with the level of immune cell infiltration.

**Figure 11:**
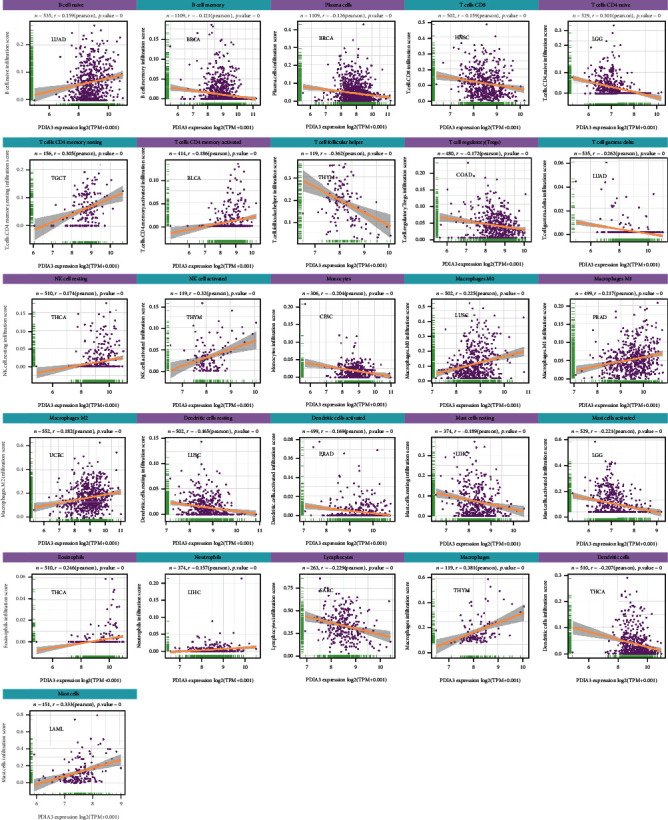
Correlation between PDIA3 expression and each immune cell infiltration level in each tumor.

At the same time, the results showed that in most types of tumors, the expression of PDIA3 was most correlated with the infiltration level of 11 kinds of immune cells. B cell memory was negatively correlated with PDIA3 expression in 14 tumors and positively correlated with PDIA3 expression in LIHC and THCA. Plasma cells were negatively correlated with PDIA3 expression in 11 tumors and positively correlated with PDIA3 expression in LUAD. T cell CD4 naive was negatively correlated with PDIA3 expression in 9 tumors and positively correlated with PDIA3 expression in HNSC, KIRP, and THCA. T cell CD4 memory resting was negatively correlated with PDIA3 expression in BRCA, LAML, and UVM and positively correlated with PDIA3 expression in 8 tumors. T cell regulatory (Tregs) was negatively correlated with PDIA3 expression in 12 tumors and positively correlated with PDIA3 expression in ESCA, GBM, KICH, LGG, LIHC, and PCPG. NK activated was negatively correlated with PDIA3 expression in six tumors and positively correlated with PDIA3 expression in BLCA, CESC, COAD, LUAD, and THYM. Macrophages M1 was positively correlated with PDIA3 expression in 14 tumors and negatively correlated with PDIA3 expression in LUAD and LUSC. Mast cell resting was negatively correlated with PDIA3 expression in 10 tumors and positively correlated with PDIA3 expression in COAD, KICH, LAML, PRAD, THCA, and THYM. Lymphocytes were negatively correlated with PDIA3 expression in 12 tumors and positively correlated with PDIA3 expression in LUAD and UVM. Macrophages were positively correlated with PDIA3 expression in 14 tumors and negatively correlated with PDIA3 expression in ESCA. Mast cells were negatively correlated with PDIA3 expression in six kinds of tumors and positively correlated with PDIA3 expression in KICH, LAML, LUSC, THCA, and THYM. The expression of PDIA3 is most significantly related to the infiltration level of immune cells in tumors (Figure 11), and the results for other cancers are shown in Supplementary Figures s2–Figure s9. In conclusion, the results of this study suggest that PDIA3 may affect the occurrence, prognosis, and treatment of a variety of cancers through its association with 11 immune cells, such as B-cell memory, plasma cells, and T-cell CD4 naive.

**Figure 12:**
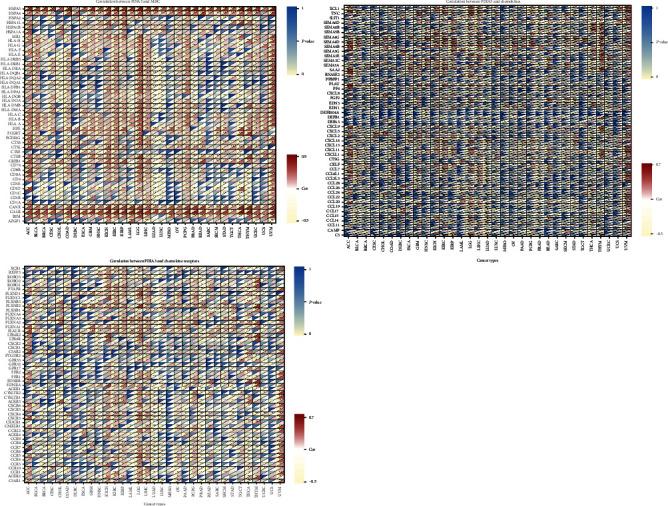
Coexpression analysis of PDIA3 expression and immune-related genes (histocompatibility complex, chemokine, and chemokine receptor protein) (^∗^*P* < 0.05, ^∗∗^*P* < 0.01, and ^∗∗∗^*P* < 0.001).

In addition, the coexpression of PDIA3 gene and immune-related genes in 33 tumors was analyzed in the study, including histocompatibility complex (MHC), chemokines, and chemokine receptor proteins. The heat map results showed that almost all immune-related genes were coexpressed with PDIA3, and these immune-related genes were negatively correlated with PDIA3 expression in most tumors (Figure 12).

### 3.8. Relationship between PDIA3 Expression and DNA Methylation

**Figure 13:**
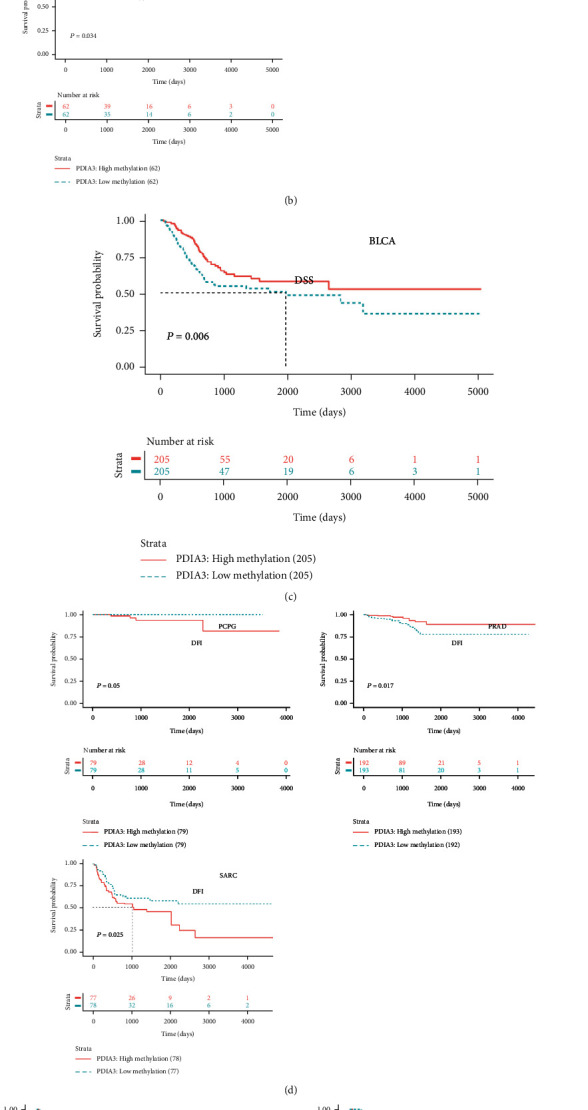
(a) Correlation between PDIA3 expression and gene promoter methylation in each tumor. (b) Correlation between PDIA3 methylation level and OS. (c) Correlation between PDIA3 methylation level and DSS. (d) Correlation between PDIA3 methylation level and DFI. (e) Correlation between PDIA3 methylation level and PFI.

In this study, HM450 methylation data in UCSC Xena database were used to analyze the correlation between PDIA3 expression and gene promoter methylation in 33 cancers. The results showed that in CESC, HNSC, and UCEC, PDIA3 expression was positively correlated with the methylation level in its promoter region, while in STAD, PDIA3 expression was negatively correlated with the methylation level in its promoter region (Figure 13(a)).

Further, Kaplan-Meier method was used to analyze the relationship between the methylation of PDIA3 promoter region and the prognosis of 33 tumor patients. The OS and the methylation of PDIA3 promoter region were analyzed, and the results showed that in BLCA, BRCA, KICH, and THYM, the methylation level of PDIA3 promoter region was the protective factor of overall survival rate, while in LIHC, the methylation level of PDIA3 was negatively correlated with OS, and the higher the methylation level of PDIA3, the worse the prognosis (Figure 13(b)). The analysis result of DSS outcome indicators showed that the methylation level of PDIA3 promoter in BLCA was the protective factor of DSS (Figure 13(c)). The analysis results of DFI outcome indicators showed that the methylation level of PDIA3 promoter was negatively correlated with DFI in PCPG and SARC and positively correlated with DFI in PRAD (Figure 13(d)). The analysis results of PFI outcome indicators showed that PDIA3 methylation level was positively correlated with PFI in BLCA and PRAD, but negatively correlated with PFI in PCPG and SARC (Figure 13(e)).

### 3.9. GO, KEGG, and GSVA Analysis

**Figure 14:**
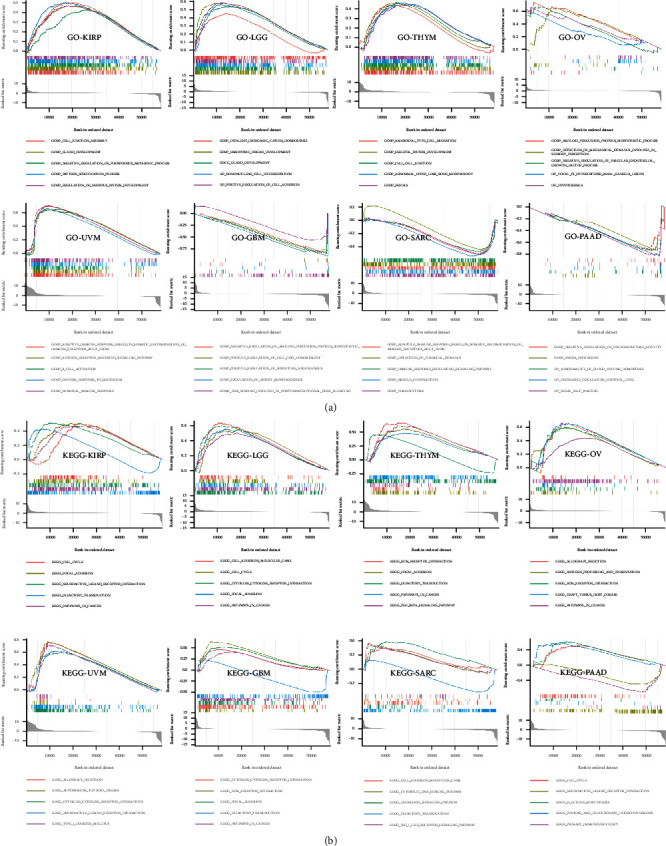
(a) GO function annotation of PDIA3 in different cancers. (b) KEGG pathway analysis of PDIA3 in different cancers. Different colored curves show different functions or pathways of regulation in different cancers. The peak value on the rising curve indicates positive regulating, and the peak value on the falling curve indicates negative regulating.

To further explore the molecular mechanism of PDIA3's involvement in regulation in different tumors, GO functional annotation and KEGG pathway enrichment analysis were performed in this study. The results showed that PDIA3 positively regulated cellular adhesion, intercellular connectivity, glandular development, and immune-related functions in KIRP, LGG, THYM, OV, and UVM, while in GBM, SARC, and PAAD, PDIA3 negatively regulated posttranscriptional gene silencing, protein synthesis, immune regulation, and vascular remodeling (Figure 14(a)). In KIRP, LGG, THYM, OV, UVM, GBM, SARC, and PAAD, PDIA3 was positively correlated with allograft rejection, cytokine adhesion, cell cycle, extracellular matrix receptor interactions, and tumor-related pathways while it was negatively correlated with olfactory pathway in GBM, KIRP, SARC, and THYM (Figure 14(b)).

**Figure 15:**
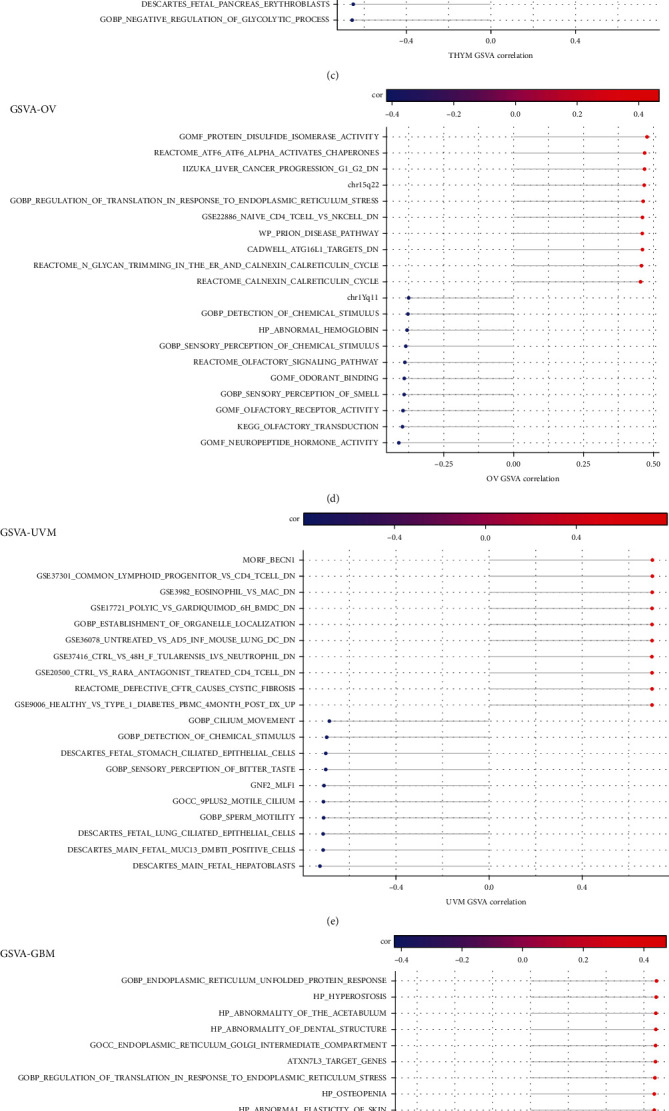
GSVA analysis of PDIA3 in each tumor. (a) KIRP. (b) LGG. (c) THYM. (d) OV. (e) UVM. (f) GBM. (g) SARC. (h) PAAD.

After further GSVA analysis, the first ten positive correlation pathways and the last ten negative correlation pathways significantly related to PDIA3 were screened out. The results showed that PDIA3 expression was closely related to immune cell pathways in LGG, OV, PAAD, SARC, and UVM. In GBM and OV, the expression of PDIA3 was positively correlated with the regulatory pathway of translation in endoplasmic reticulum stress response. In KIRP and OV, PDIA3 expression was involved in glycan reaction process and calcium cycle process. In PAAD and KIRP, PDIA3 expression was positively correlated with PSMC2 protein encoding gene, and PDIA3 was involved in regulating cell proliferation and differentiation, apoptosis, and signal transduction (Figure 15).

## 4. Discussion

The results of this study showed that the PDIA3 gene was highly expressed in 19 types of tumor tissues and only lowly expressed in thyroid cancer tumor tissues. These results were consistent with the trend of IHC analysis. Many previous studies have also shown that PDIA3 is expressed in various tumors such as gastric cancer, prostate cancer, kidney cancer, melanoma, cervical cancer, ovarian cancer, breast cancer, lung cancer, esophageal squamous cell carcinoma, colon cancer, liver cancer, and throat cancer, and it is associated with tumor progression, metastasis, and patient survival prognosis [14–17].

The results of survival analysis in this study show that the high expression of PDIA3 in CESC, HNSC, LGG, LUAD, KIRP, KICH, and UVM is correlated with the poor patient prognosis. In contrast, high PDIA3 expression is associated with good prognosis in patients with UCEC, OV, PRAD, and THCA; the results of OS, DSS, DFI, and PFI showed that PDIA3 was a high-risk factor for KIRP; and the results of OS, DSS, and PFI analysis were highly consistent, indicating that PDIA3 was the high-risk gene with the greatest risk of KICH. Previous studies have shown that the downregulation of PDIA3 is related to the poor prognosis of early cervical cancer, and PDIA3 expression shows significant differences in different histological types of cervical cancer, which has a direct impact on drug therapy and clinical application and can be regarded as a potential and specific prognosis and therapeutic target of cervical cancer [18–20]. The study of He et al. [21] showed that the expression level of PDIA3 protein showed an increasing trend from normal mucosa to early stage of oral squamous cell carcinoma, and it could be used as a potential biomarker to assist the diagnosis of oral squamous cell carcinoma.

The research results of Zou et al. [22] showed that the high expression of PDIA3 played an important role in the progression of glioma, which can predict the survival outcome and treatment response of glioma patients. On the contrary, the high expression of PDIA3 in UCEC and THCA was related to good patient prognosis. The study results of Kure et al. showed that PDIA3 expression in thyroid tumor tissue was significantly lower than that in normal thyroid tissue. Compared with patients with high expression of PDIA3, patients with low expression of PDIA3 showed worse cause-specific survival [23]. The study of Shimoda et al. [24] showed that PDIA3 expression was an independent factor of tumor staging in gastric cancer patients, and the overall survival time of PDIA3-high cases was significantly better than that of PDIA3-low cases, especially in advanced cases.

In addition, this study found that PDIA3 expression was related to the patient's age in some tumors. In LAML, LIHC, PRAD, and READ, the expression level of PDIA3 was higher in the middle and low age groups, but in SARC and STAD, it was higher in the middle and high age groups. It has been reported [25] that PDIA3 siRNA can effectively promote the apoptosis of AML cells and inhibit the proliferation, invasion, and migration of AML cells by regulating oxidative phosphorylation, amino sugar and nucleotide sugar metabolism pathway, and MAPK signaling pathway, thus providing a new therapeutic target for AML. These results will play a guiding role in the later development of individualized treatment strategies for patients in different age groups. In this study, after the relationship between PDIA3 and clinical stages was analyzed, it was found that PDIA3 expression was statistically significant in different clinical stages of five tumors, such as BLCA, KICH, KIRC, LUSC, and THCA. Leys et al. [26] studied the expression of PDIA3 in 164 patients with gastric cancer. The results showed that there was a significant difference in its expression between normal gastric mucosa and gastric gland cancer cells in primary and metastatic tumors, and the low expression of PDIA3 in tumors was indeed related to the increase of tumor invasion depth, the advance of overall clinical stage of disease, and the decrease of postoperative survival rate. Yang et al. [27] studied PDIA3 expression in renal cell carcinoma, and its expression was significantly higher than that of matched adjacent renal cell carcinoma tissues. Therefore, PDIA3 may become a new candidate marker for the diagnosis of renal cell carcinoma, which provides important significance for early clinical diagnosis of renal cell carcinoma. These results clearly indicate that PDIA3 can be used as a biomarker to determine the prognosis of various cancers.

Immunotherapy based on immune checkpoint inhibitors significantly improved the objective remission rate and overall survival time of patients with advanced malignant tumors, but not all patients benefited from immunotherapy. Therefore, it is necessary to find better predictive markers. Tumor mutation load (TMB) can indirectly reflect the ability and degree of tumor to produce new antigens, and it has been proved that it can predict the immunotherapy efficacy of many kinds of tumors [28, 29]. Microsatellite instability (MSI) is also an important marker molecule of immune checkpoint inhibitors, which has a predictive role in tumor immunotherapy.

In this study, it is found that PDIA3 expression was related to TMB in 13 tumors and MSI in 9 tumors. Among them, the expression level of PDIA3 in THYM has the strongest correlation with TMB, and the expression level of PDIA3 in READ has the strongest correlation with MSI. These results show that the expression level of PDIA3 will affect the TMB and MSI of tumors, thus affecting the response of patients to immunotherapy. At the same time, the relationship between PDIA3 and tumor immune microenvironment was also discussed. The results showed that PDIA3 was negatively correlated with immune score and matrix score in 6 tumors. Among them, in LGG, the expression level of PDIA3 has the strongest correlation with immune score and matrix score, showing a significant positive correlation. It was confirmed that the expression of PDIA3 was closely related to the biological process of LGG immune cells and immune-related molecules. Further analysis of the relationship between PDIA3 and tumor immune cell infiltration showed that PDIA3 expression was related to immune cell infiltration in most types of tumors, especially immune cells such as B cell memory, plasma cells, CD4 naive T cells, memory resting T cells CD4, Tregs, activated NK cell, M1 macrophages, macrophages, and mast cells. Studies have shown that PDIA3 plays a key role in maintaining the antitumor immune response, which may become a potential cancer treatment target, used for the treatment of tumors that previous treatments have failed to induce strong T cell-mediated immune response. PDIA3 is indispensable in antigen processing and presentation, contributing to the activity of T cell-mediated immune responses [30]. PDIA3 expression is related to CD8+ cytotoxic T lymphocyte dysfunction. When PDIA3 is knocked out in human CD8+ T cells, the antitumor activity of CD8+ T cells is enhanced, because the knockout of PDIA3 in CD8+ T cells regulates a variety of immunomodulatory factors and effector factors on the cell surface [31]. PDIA3 induces the death of immunogenic cells in chemotherapy cells, which is essential for maintaining immunogenicity. PDIA3 may be a potential indicator of the efficacy of cancer immunotherapy [32]. Transcriptome analysis, single cell sequencing, cytokine analysis, and T cell signal transduction analysis show that editing PDIA3 in T cells can enhance the effector function, and engineered PDIA3 mutant EGFRvIII chimeric antigen T cells are more effective in antigen-specific killing of human glioblastoma cells. Chiavari et al. [33] showed that the decrease of PDIA3 expression and activity in glioblastoma cells significantly restricted the tumor-loving polarization of microglia to M2 phenotype and the production of proinflammatory factors. These results suggest that PDIA3 expression is closely related to tumor immune cell infiltration in most cancers, affecting the prognosis of patients, and LGG is the tumor most likely to be affected by the tumor microenvironment to affect its development and prognosis.

At present, there are few studies on methylation level of PDIA3 promoter region and tumorigenesis. In this study, the relationship between PDIA3 expression and methylation level of promoter region was systematically analyzed. The results showed that PDIA3 expression had positive correlation with methylation level of promoter region in CESC, HNSC, and UCEC, but negative correlation in STAD. The study of Abdula et al. [34] showed that the overall methylation level of PDIA3 gene promoter in Uyghur female cervical cancer was higher than that in precancerous lesions and normal control tissues, but hypermethylation only occurred in specific CpG islands and sites. At the same time, the relationship between PDIA3 promoter region methylation and patient outcome was further analyzed, and it is found that PDIA3 promoter region methylation level was a protective factor for survival in some tumors, especially in BLCA, and PDIA3 promoter region methylation level was positively correlated with OS, DSS, and PFI. These results opened up a new direction and way for us to study the relationship between PDIA3 methylation and tumor occurrence and development later.

In this study, the GO and KEGG enrichment and GSVA of PDIA3 were further analyzed. The results showed that PDIA3 may affect the tumorigenesis and pathological process by affecting cell adhesion, protein synthesis, vascular remodeling, cell cycle, and immune function. These data are consistent with many previous research results, i.e., the role of PDIA3 in regulating T cell-mediated immune response, immunogenic cell death, UPR, DNA repair signal pathway, and membrane activation signal pathway [35, 36].

In conclusion, the pan-cancer analysis of PDIA3 in this study shows the correlation between the differences of PDIA3 in tumor tissues and normal tissues and clinicopathological features. The results of this study show that PDIA3 can be used as an independent prognostic factor to predict the prognosis of various tumors, especially, it plays an important role in the prognosis of KIRP and KICH patients. Meanwhile, PDIA3 expression is closely related to immune-related factors such as TMB, MSI, MMR, and TME in various tumors. In particular, it has an important impact on the immunotherapeutic response of patients with THYM, READ, and LGG and highly correlated with the expression level of immune infiltrating cells, which has different effects on immunogenicity in different tumors. This study reveals the expression and role of PDIA3 gene as a biomarker for immunotherapy and prognosis in various cancers with the view to strengthen researchers' understanding of PDIA3 and provide new ideas and directions for PDIA3 to become a new clinical biomarker.

## Data Availability

The data that support the findings of this study are available from the corresponding author upon reasonable request.

## Supplementary Materials

Supplementary Materials Fig s1: PDIA3 between tumor tissues and normal tissues in TCGA database for other cancers. Fig s2-s9: correlation between PDIA3 expression and each immune cell infiltration level in other cancers.
