# Supplementary material for: Pan-Cancer Analysis of PDIA3: Identifying It as a Potential Biomarker for Tumor Prognosis and Immunotherapy
Source: Oxid Med Cell Longev. 2022 Aug 22;2022:9614819. doi: 10.1155/2022/9614819 (PMC9423987; doi:10.1155/2022/9614819)
Supplement: Supplementary Materials — Fig s1: PDIA3 between tumor tissues and normal tissues in TCGA database for other cancers. Fig s2-s9: correlation between PDIA3 expression and each immune cell infiltration level in other cancers. [file 9614819.f1.docx]

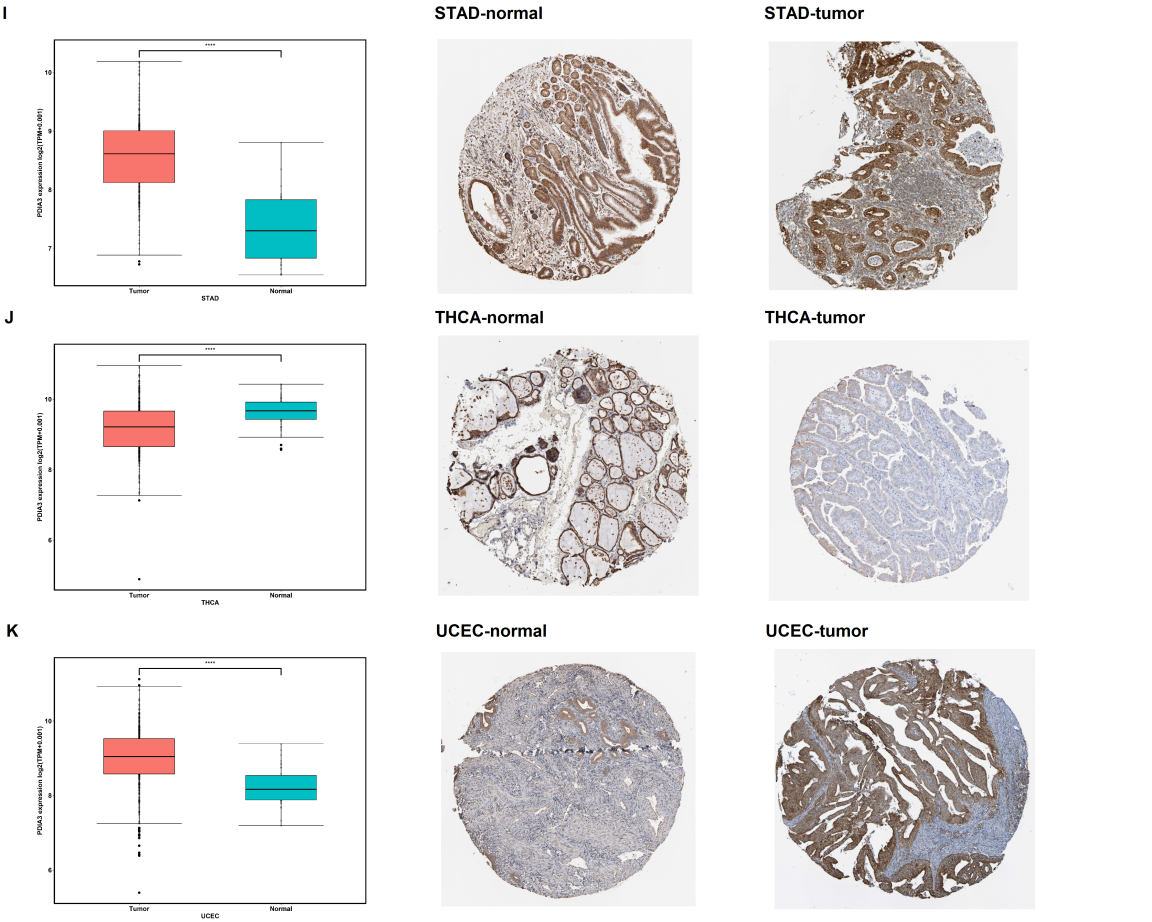


Figure s1. PDIA3 between tumor tissues and normal tissues in TCGA database for other cancers


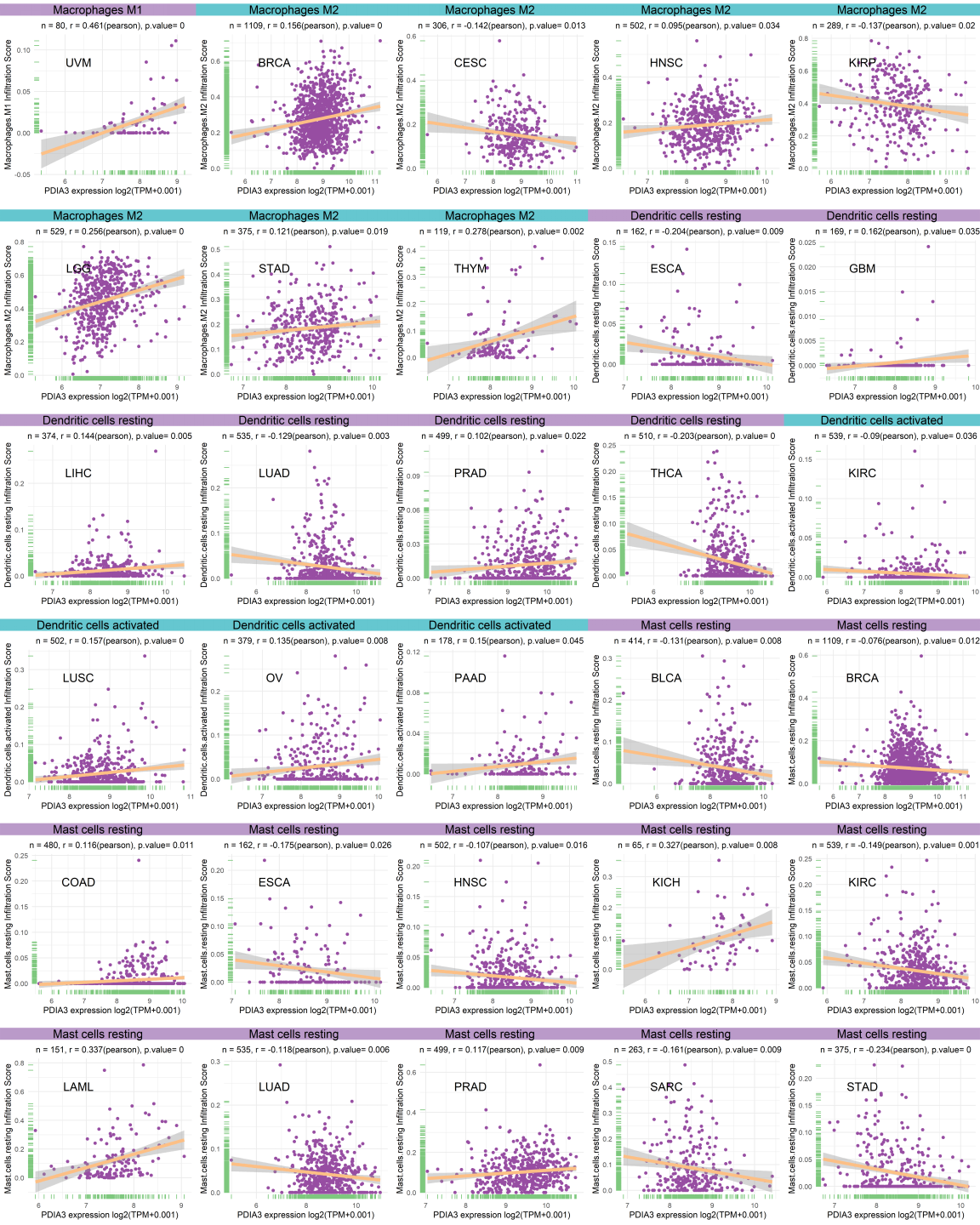

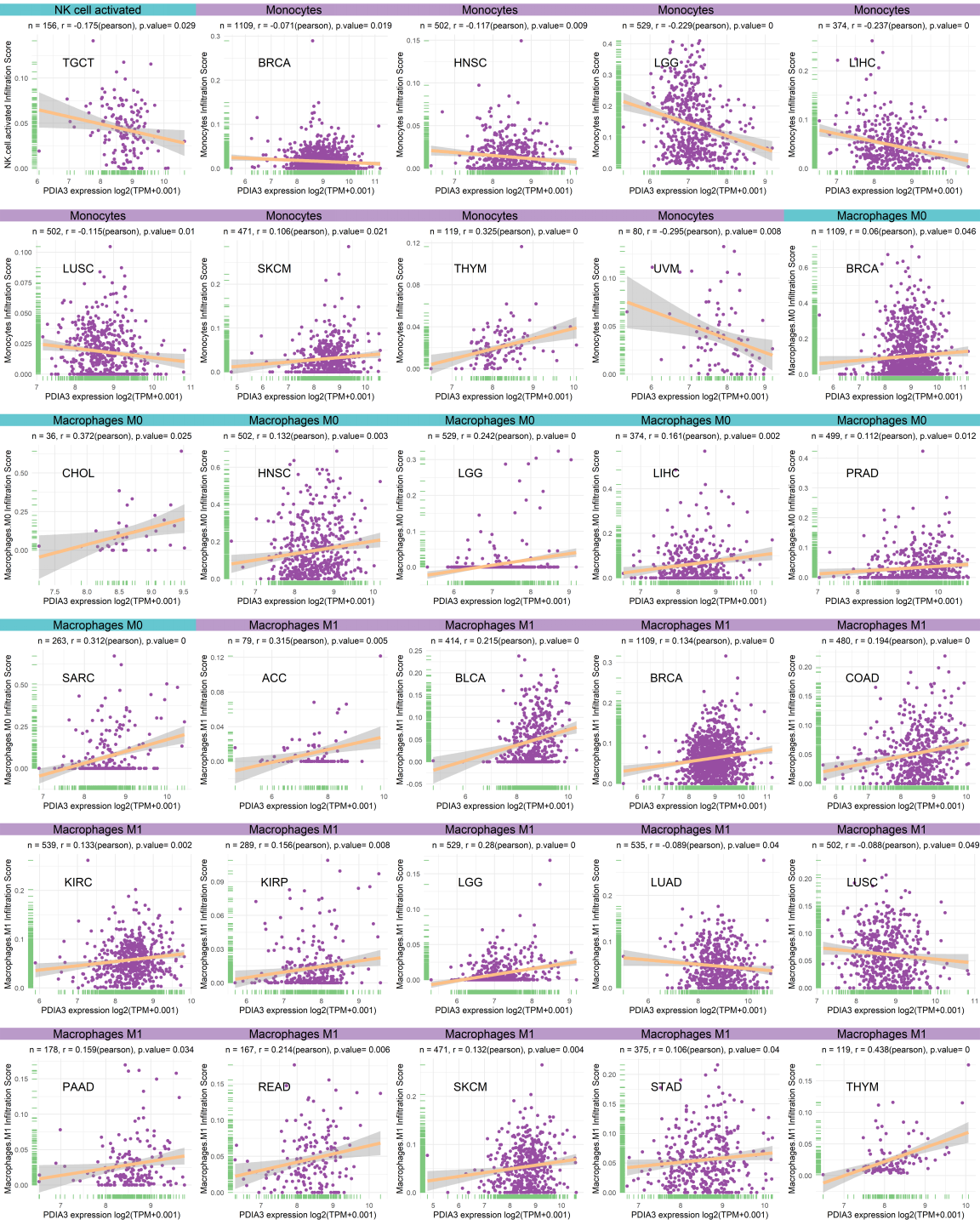

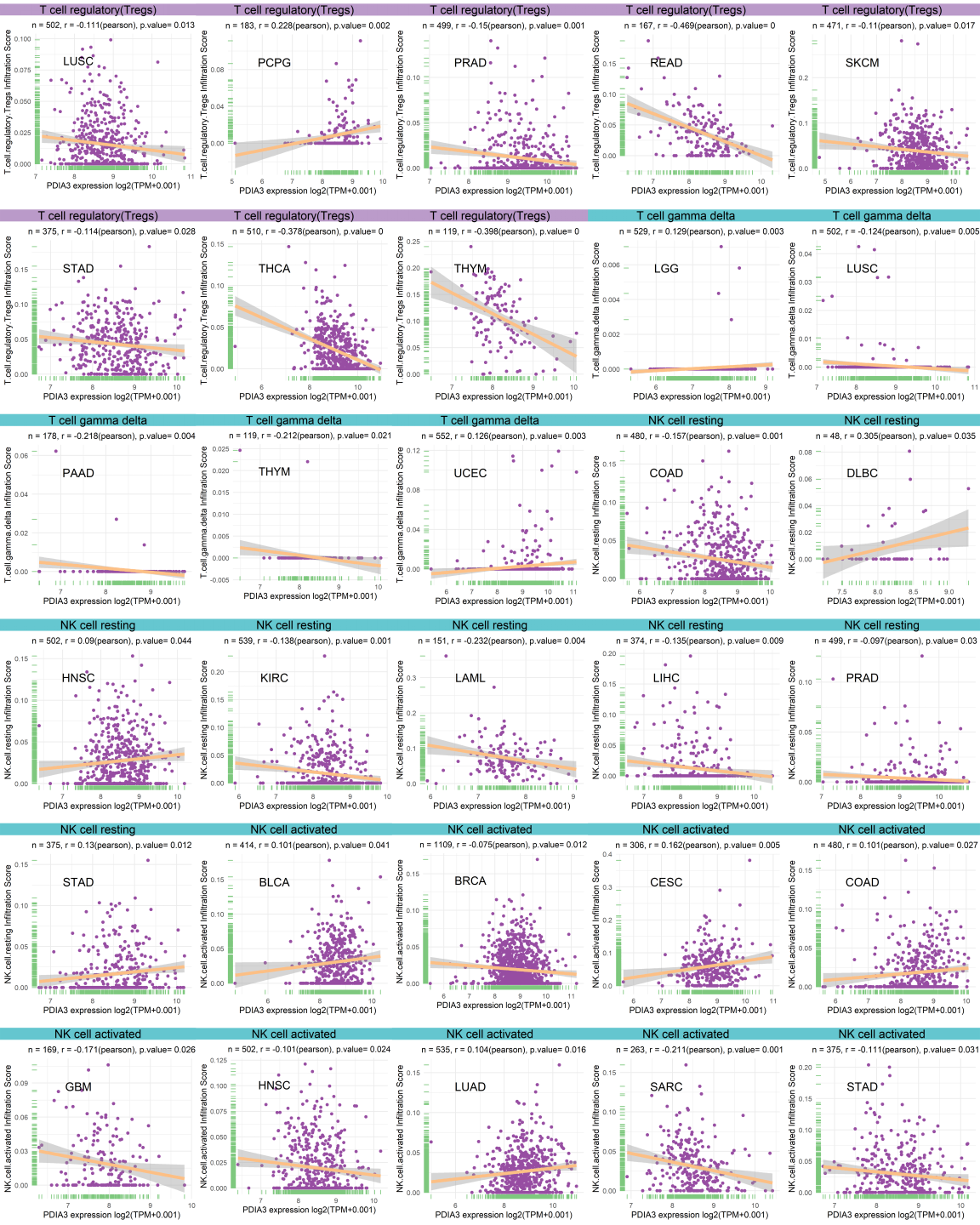

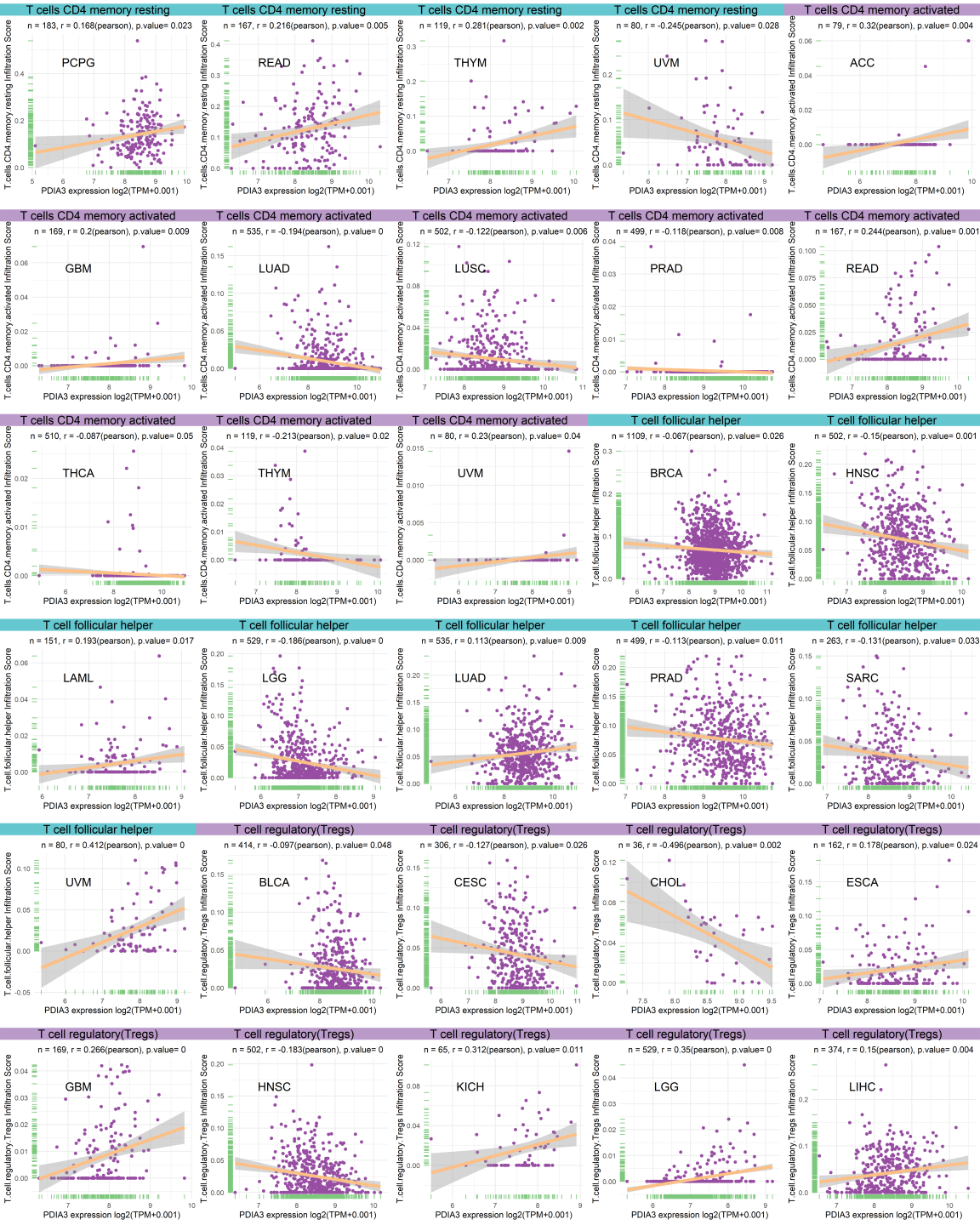

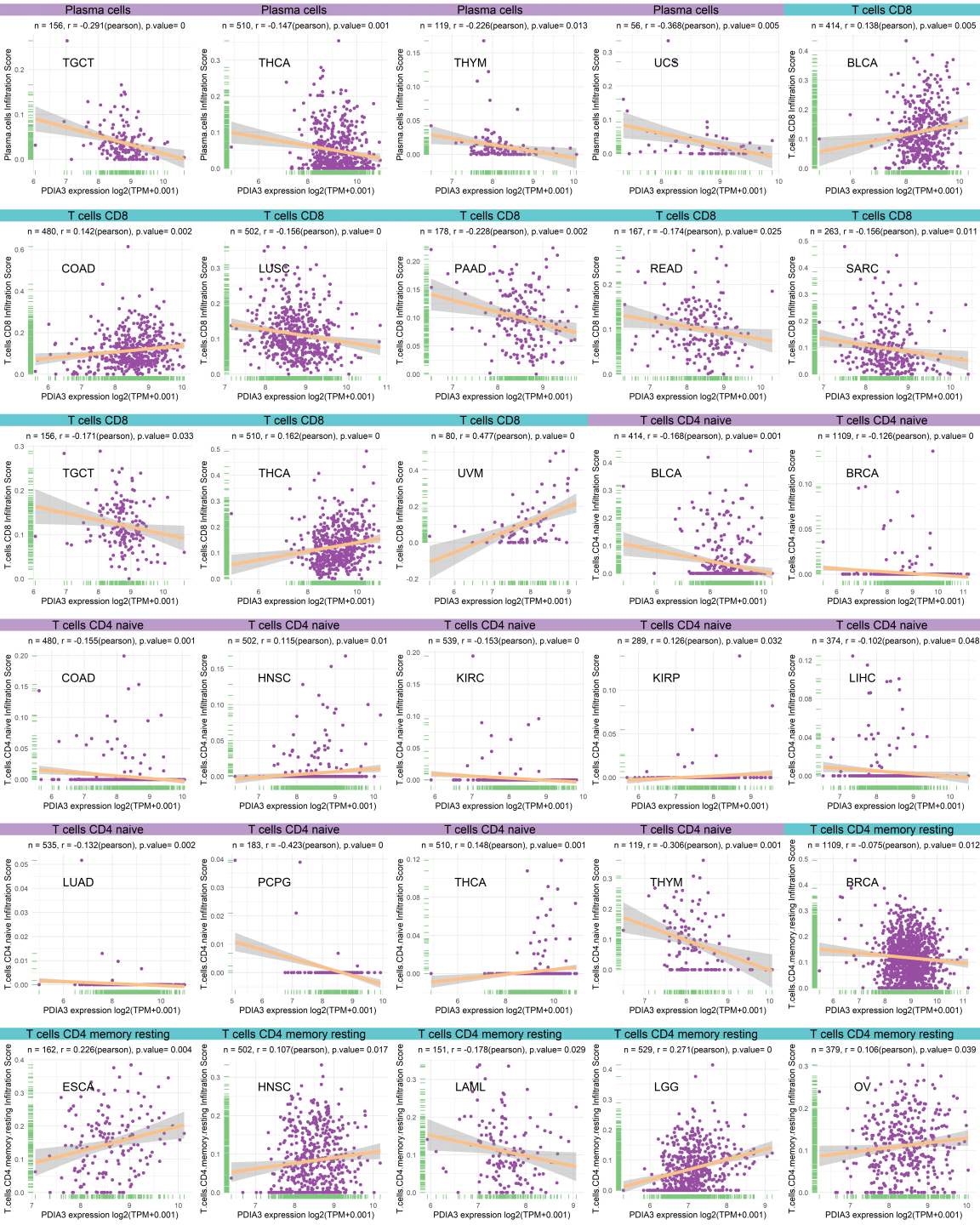

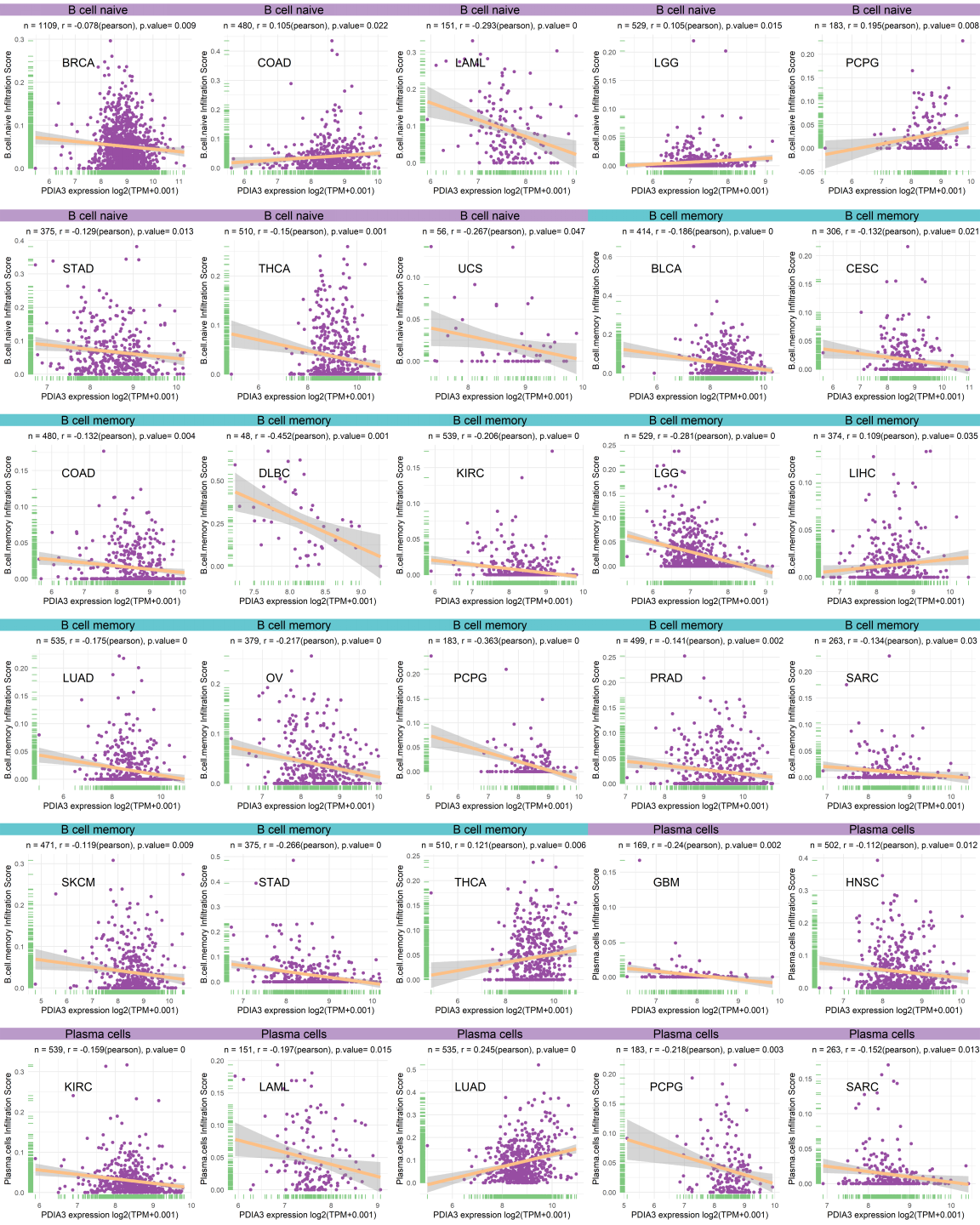

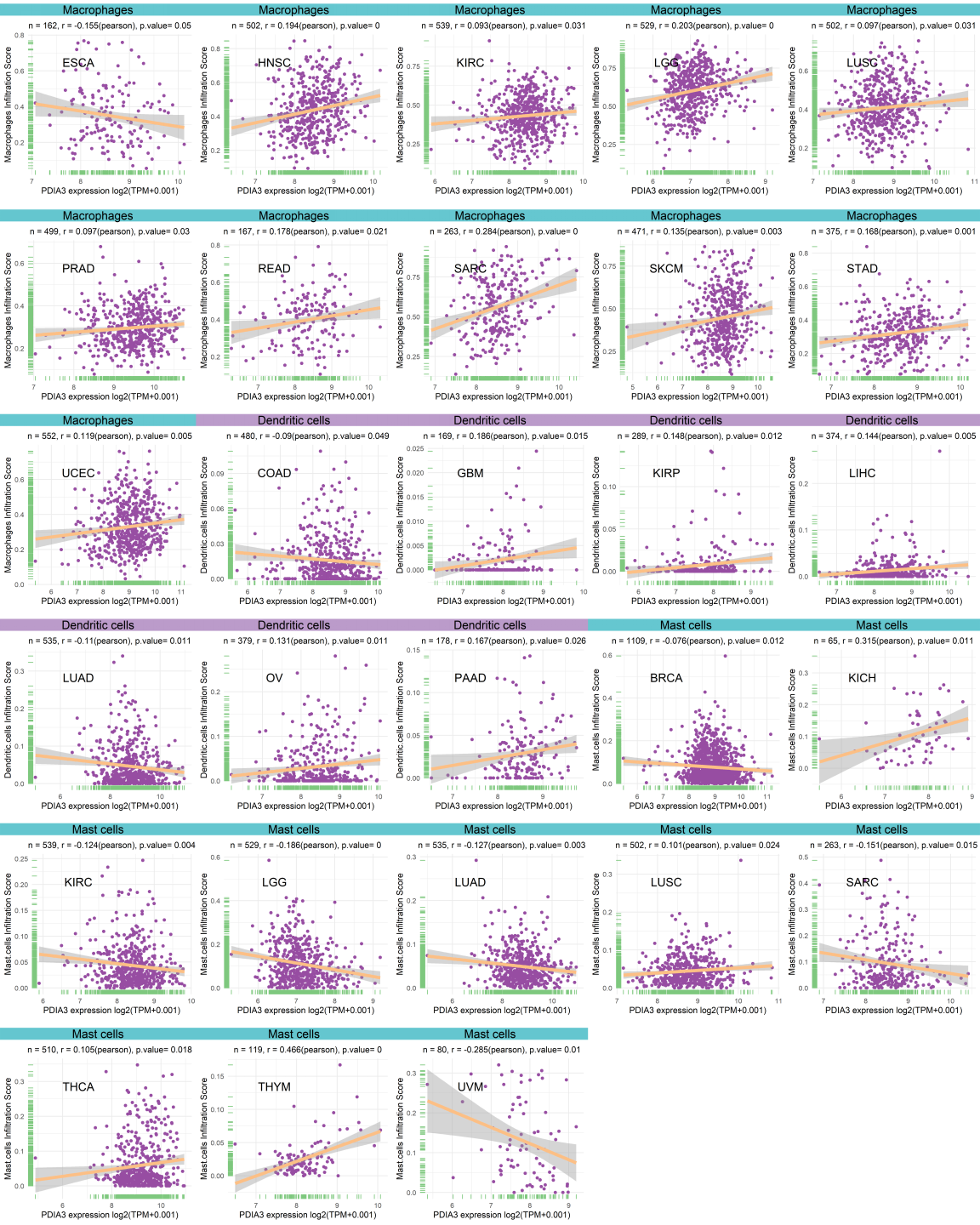

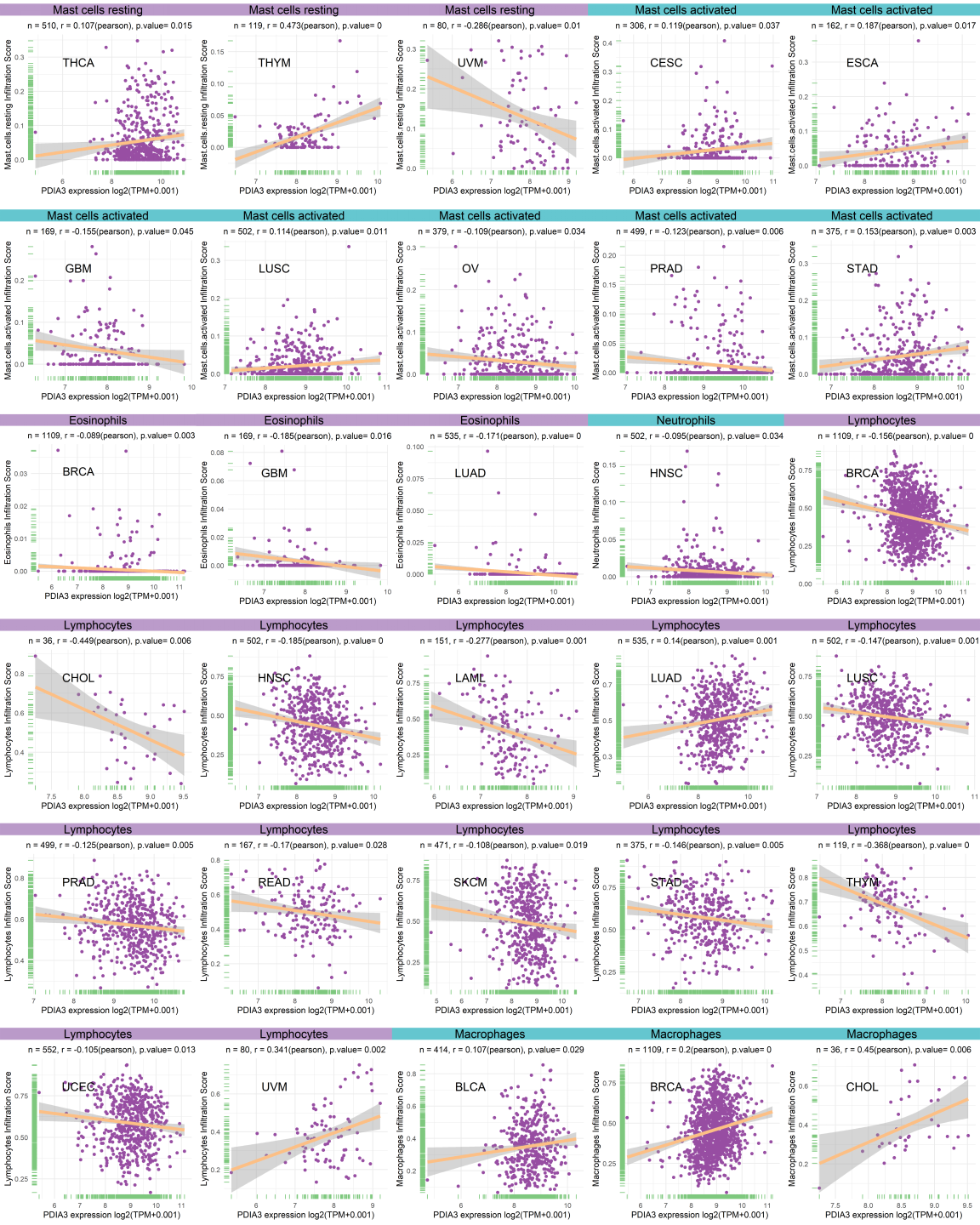


Figure s2-s9. Correlation between PDIA3 expression and each immune cell infiltration level in other cancers
